# Magnesium Ions Moderate Calcium-Induced Calcium Release in Cardiac Calcium Release Sites by Binding to Ryanodine Receptor Activation and Inhibition Sites

**DOI:** 10.3389/fphys.2021.805956

**Published:** 2022-01-25

**Authors:** Bogdan Iaparov, Iuliia Baglaeva, Ivan Zahradník, Alexandra Zahradníková

**Affiliations:** Department of Cellular Cardiology, Institute of Experimental Endocrinology, Biomedical Research Center of the Slovak Academy of Sciences, Bratislava, Slovakia

**Keywords:** cardiac myocyte, cardiac dyad, calcium spark, ryanodine receptor, calcium release site

## Abstract

Ryanodine receptor channels at calcium release sites of cardiac myocytes operate on the principle of calcium-induced calcium release. *In vitro* experiments revealed competition of Ca^2+^ and Mg^2+^ in the activation of ryanodine receptors (RyRs) as well as inhibition of RyRs by Mg^2+^. The impact of RyR modulation by Mg^2+^ on calcium release is not well understood due to the technical limitations of *in situ* experiments. We turned instead to an *in silico* model of a calcium release site (CRS), based on a homotetrameric model of RyR gating with kinetic parameters determined from *in vitro* measurements. We inspected changes in the activity of the CRS model in response to a random opening of one of 20 realistically distributed RyRs, arising from Ca^2+^/Mg^2+^ interactions at RyR channels. Calcium release events (CREs) were simulated at a range of Mg^2+^-binding parameters at near-physiological Mg^2+^ and ATP concentrations. Facilitation of Mg^2+^ binding to the RyR activation site inhibited the formation of sparks and slowed down their activation. Impeding Mg-binding to the RyR activation site enhanced spark formation and speeded up their activation. Varying Mg^2+^ binding to the RyR inhibition site also dramatically affected calcium release events. Facilitation of Mg^2+^ binding to the RyR inhibition site reduced the amplitude, relative occurrence, and the time-to-end of sparks, and vice versa. The characteristics of CREs correlated dose-dependently with the effective coupling strength between RyRs, defined as a function of RyR vicinity, single-channel calcium current, and Mg-binding parameters of the RyR channels. These findings postulate the role of Mg^2+^ in calcium release as a negative modulator of the coupling strength among RyRs in a CRS, translating to damping of the positive feedback of the calcium-induced calcium-release mechanism.

## Introduction

Magnesium ions, as the most abundant divalent cations in the cell cytoplasm, take part in many cellular processes including the contraction-relaxation cycle and excitation-contraction coupling of cardiac myocytes. They often act in antagonism to the activating function of calcium. In excitation-contraction coupling, the Mg/Ca antagonism arises from the direct and indirect interactions of Mg^2+^ with the ryanodine receptor (RyR). Since the cytosolic concentration of free Mg^2+^ is by several orders of magnitude over that of Ca^2+^, magnesium ions compete effectively with calcium ions for binding to the RyR calcium-activation site despite their relatively low affinity (Rousseau et al., 1986; Xu et al., 1996; Laver et al., 1997; Copello et al., 2002; Zahradnikova et al., 2003). Additionally, the ability to bind polyphosphates makes Mg^2+^ an important regulator of ATP-dependent processes through buffering the free ATP concentration and thus plays indirectly a role in the priming of RyRs by ATP to activation by Ca^2+^ (Xu et al., 1996; Tencerova et al., 2012).

In dyads of cardiac myocytes, the activation of RyR channels causes efflux of Ca^2+^ ions to the cytosol that can be observed as a transient and localized increase of Ca^2+^ concentration, dubbed calcium spark (Cheng and Lederer, 2008). In the absence of external activating stimulus, the activation of RyRs may happen by chance due to the non-zero RyR open probability at the resting cytosolic Ca^2+^ concentration and lead to the production of spontaneous calcium sparks (Cheng and Lederer, 2008; Zahradnikova et al., 2010) as well as of the non-spark (invisible) calcium release flux (Niggli and Shirokova, 2007; Bovo et al., 2011; Shang et al., 2014). Both the sparks and non-sparks are of small amplitude and duration and thus many of them are buried in the background noise. Still, the relationship between the frequency, amplitude, and time course of calcium release events (CREs) and the activity of RyR channels *in situ* has not been determined due to experimental difficulties. Analysis of observable calcium sparks indicated simultaneous activation of many (Bridge et al., 1999; Lukyanenko et al., 2000) or only a few RyRs (Wang et al., 2004; Janicek et al., 2012). These findings are inconsistent with the calcium-induced calcium-release mechanism of cardiac excitation-contraction coupling (Fabiato, 1983; Stern, 1992). The reason is most likely in the complex regulation of RyR activity that depends on many cytosolic factors (Meissner, 1994, 2004). Among these, the Ca/Mg antagonism is likely to play a central role.

The allosteric nature of the interaction between the activation sites of the RyR and the closed-open transition (Zahradnik et al., 2005; Zahradnikova et al., 2010) has been inferred from single-channel data of mutant RyR2 (Li and Chen, 2001) and the calcium dependence of calcium spark frequency (Lukyanenko and Gyorke, 1999). The allosteric mechanism was confirmed by the effect of ligands on the structure of RyR channels (des Georges et al., 2016; Chi et al., 2019). It was found that the conformation of the Ca^2+^ binding site affects the closed - open transition of the pore: the conformation with a low affinity for Ca^2+^ promotes the closed state of the pore, while the conformation with a high affinity for Ca^2+^ promotes the open state of the pore (Dashti et al., 2020).

Mechanisms by which Mg^2+^ affects RyR open probability were revealed by single-channel experiments. Inhibition of RyR activity by Mg^2+^ partially persisted at high cytosolic Ca^2+^ concentration, indicating the presence of a RyR inhibitory site (Laver et al., 1997; Zahradnikova et al., 2003). At the same time, the inhibition could be partially overcome by elevated Ca^2+^ levels, indicating competition between Ca^2+^ and Mg^2+^ binding at the RyR activation site (Laver et al., 1997; Zahradnikova et al., 2003).

The inhibitory effect of Mg^2+^ on ryanodine receptors was observed also in cardiac myocytes as a transient decrease of the frequency of sparks upon an increase in cytosolic free Mg^2+^ concentration (Lukyanenko et al., 2001; Gusev and Niggli, 2008). This process could not be studied in more detail because of rapid changes in the SR calcium load occurring in response to changes in calcium release,

A partial insight into the dynamics of RyR activation *in situ* was provided by two recent models of the calcium release site (CRS) based on RyRs with the same, realistic calcium dependence of the steady-state open probability in the presence of Mg^2+^ but with different kinetic assumptions. Postulating binding of Ca^2+^ ions to the RyR activation site as instantaneous and unbinding of Mg^2+^ from the calcium activation site as slow predicted 2-3 simultaneously open RyR channels at the peak of a spark (Zahradnikova and Zahradnik, 2012). Considering the equilibration between Ca^2+^ and Mg^2+^ on the RyR activation site as instantaneous predicted three types of calcium release events differing in the number of open RyRs (Iaparov et al., 2021): CREs consisting of only a single RyR opening that did not activate nearby RyRs in a CRS (quarks); CREs that involved activation of a small subset of RyRs and simultaneous opening of 2-3 RyRs in a CRS (blip); and CREs that involved sequential activation of all RyRs in the CRS and simultaneous opening of about half of the RyRs in the CRS (sparks). This *in silico* study indicated the role of dynamic Ca^2+^ and Mg^2+^ interaction with RyRs of a calcium release site that can be solved only by a RyR model explicitly incorporating the respective rate constants.

The need for a better understanding of the role of Mg^2+^ in the behavior of cardiac dyads is underlined by the findings that the aberrant behavior of RyRs in diseased myocytes may be in part due to a changed interaction of RyRs with Mg^2+^. Several central domain RyR mutations causing calcium handling-related catecholaminergic polymorphic ventricular tachycardia (CPVT) showed a decreased inhibition of RyR activity by Mg^2+^ ions (Lehnart et al., 2004; Guo et al., 2020). Aberrant interaction with Mg^2+^ in the skeletal muscle isoform RyR1 due to mutations has been also postulated as an important cause of malignant hyperthermia, the skeletal muscle analog of CPVT (Steele and Duke, 2007). However, it is not known whether the changes in Mg^2+^-RyR interaction are due to a decreased competition between Mg^2+^ and Ca^2+^ on the calcium activation site, due to a weaker Mg^2+^ interaction with the inhibition site, or both. Moreover, in heart failure of various etiologies, RyR2 hyper-phosphorylation has been observed in parallel with increased RyR open probability, calcium sensitivity, spark frequency, or diastolic calcium release (Marx et al., 2000; Okuda et al., 2018), and these effects were at least in part caused by changes in Mg^2+^ regulation of RyRs (Li et al., 2013).

Cardiac diseases and RyR mutations induce a variety of changes in calcium release. In heart failure (HF), increased non-spark leak, increased spark size, and decreased spark frequency were observed (Kolstad et al., 2018); in atrial fibrillation (AF), increased duration, frequency, and time to peak of sparks were observed (Macquaide et al., 2015). The changes observed at the cellular level might stem not only from changes in RyR gating but also from a changed structure of the calcium release sites that occurred in some (Macquaide et al., 2015; Kolstad et al., 2018) but not all models of cardiac disease (Munro et al., 2021). It is not known whether the changes observed in diseased cardiac myocytes are due to changes in the action of Mg^2+^, but the RyR inhibitor dantrolene, which requires Mg^2+^ to exert its effect (Choi et al., 2017), was shown to have beneficial effects in AF (Hartmann et al., 2017). Thus, the relationship between the RyR sensitivity to Mg^2+^ and the resulting behavior of CRSs needs to be estimated. However, due to the complex relationship between RyR activity and activity of calcium release sites, this task would require complex and expensive experiments with an uncertain outcome. Instead, *in silico* modeling can provide useful insights by tests of working hypotheses, comparison of data obtained by different approaches and laboratories, and/or execution of virtual experiments that are not experimentally feasible yet.

**FIGURE 1:**
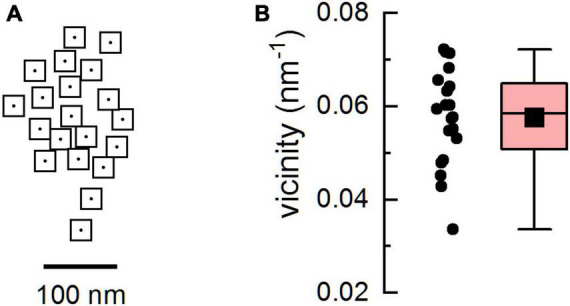
The geometric model of the calcium release site. **(A)** The arrangement of ryanodine receptors (RyRs) in the model CRS. The empty squares with dots represent RyRs with a central ion channel, the gated point source of Ca2+ current. **(B)** The distribution of RyR vicinities in the model CRS. The box diagram shows the 25% and 75% percentile, whiskers show minimum and maximum, the black square depicts the mean, and the horizontal line represents the median.

**FIGURE 2:**
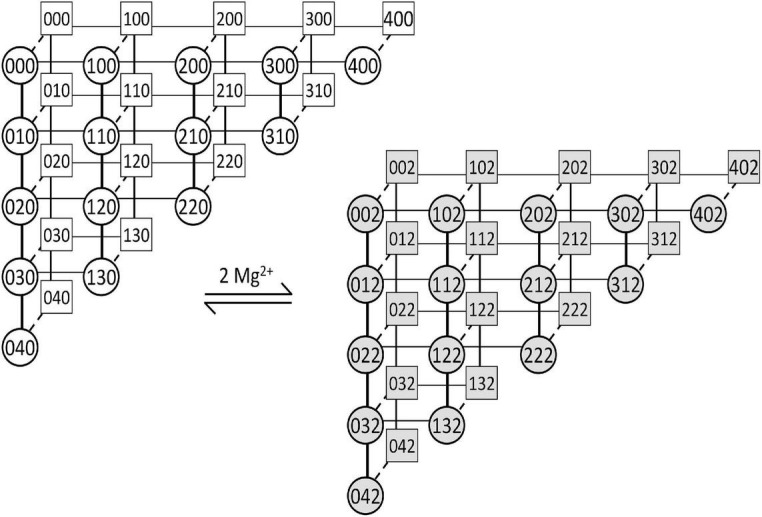
The gating scheme of the MWC-Ca/Mg RyR model. The scheme depicts transitions of four allosterically coupled monomeric activation sites and one common inhibitory site of the RyR channel. The competitive binding of Ca^2+^ and Mg^2+^ ions to RyR activation sites is shown by horizontal (thin lines) and vertical (thick lines) transitions, respectively. The RyR open/close transitions (dashed lines) are independent of ion binding/unbinding. The RyR inhibitory site binds two Mg^2+^ ions simultaneously and switches the RyR channel to a non-conducting state without affecting the activation sites. Squares – closed states; Circles – open states; Gray – inhibited states. The three numerals in the symbols denote (from left to right): the number of Ca^2+^ ions bound to the activation site; the number of Mg^2+^ ions bound to the activation site; and the number of Mg^2+^ ions bound to the inhibition site. All RyR transitions between states are reversible and obey the principle of detailed balance.

In this study, we used a model of the calcium release site (Figure 1) based on a quantitative description of RyR gating (Figure 2), calcium diffusion, and ligand binding to analyze the dependence of simulated calcium release events on parameters relevant to Mg^2+^ binding. The explored parametric space of the CRS model included the description of stochastic RyR gating that allowed binding of Mg^2+^ to the RyR magnesium inhibitory site, Mg^2+^/Ca^2+^ competitive binding to the RyR calcium-activation site, and allosteric regulation of RyR opening by Ca^2+^ and Mg^2+^ binding to the activation site. The simulated CRS activity in response to a random opening of a single RyR was analyzed for the fractions of generated quarks, blips, and sparks, as well as for the amplitudes and time courses of generated CREs. Inclusion of the kinetics of Ca- and Mg-binding to the RyR gating model allowed us to redefine the role of magnesium ions in the CRS function.

## Methods

### Simulations and Analysis

The program for performing simulations was written in C++ and run on a PC with CPU AMD Ryzen 9 3900X (12 cores, 24 threads), 32 GB RAM. Individual calcium release events were simulated in parallel using the OpenMP API^1^. Parallel generation of random numbers was performed using OMPRNG (Bognar, 2013). The program consumed up to 6 GB of RAM during simulations. Cytosolic calcium signals were calculated using CalC (Matveev et al., 2002). Simulated records of RyR activity were analyzed in Python using the libraries NumPy (Harris et al., 2020), pandas (McKinney, 2010), SciPy (Virtanen et al., 2020), scikit-learn (Pedregosa et al., 2011), and lmfit (Newville et al., 2014). Figures were prepared using OriginPRO Ver. 2020b (OriginLab, United States).

### Kinetic Parameters of the Ryanodine Receptor Gating Model

Nine of the 14 independent kinetic parameters of the RyR gating model (Table 1) were determined by approximating the published RyR single-channel characteristics obtained in bilayer experiments either from steady-state records of RyR activity (open probabilities, mean open times) or from responses of RyR activity to a laser pulse-induced stepwise [Ca^2+^] increase (activation times). These experiments were performed on RyRs in isolated cardiac microsomal vesicles incorporated into lipid bilayers. RyR single-channel activity was recorded using Cs^+^ or Cs^2+^ ions as charge carriers in the presence of variable concentrations of total ATP (0 – 3 mM), free Mg^2+^ (0 – 1.3 mM), and free Ca^2+^ (0.1 – 100 μM) at the cytosolic side and 1 mM free Ca^2+^ at the luminal side (Zahradnikova et al., 1999, 2003; Tencerova et al., 2012; Cannell et al., 2013). The corresponding kinetic parameters (Table 1) were fitted and the standard errors of the fit were estimated using the differential evolution method (Storn and Price, 1997) from lmfit (Newville et al., 2014). The remaining kinetic parameters in Table 1 were either taken directly from the literature (two parameters), or set according to known estimates (one parameter), or set to zero according to a reasonable guess (two parameters).

**TABLE 1:** Parameters of the ryanodine receptor (RyR) model and their reference values.

Parameter	Value	Unit	References
*K*_*ACa*_	4.18 ± 0.13	μM	*k*_*AoffCa*_/*k*_*AonCa*_
*k*_*AonCa*_	0.71	μM^–1^ ms^–1^	Zahradnikova et al., 1999
*k*_*AoffCa*_	2.97 ± 0.09	ms^–1^	Fitted, this study.
*K*_*AMg*_	92.3 ± 3.5	μM	*k*_*AoffMg*_/*k*_*AonMg*_
*k*_*AonMg*_	0.0071	μM^–1^ ms^–1^	0.01 × *k*_*AonCa*_, Eigen and Wilkins, 1965
*k*_*AoffMg*_	0.66 ± 0.025	ms^–1^	Fitted, this study.
*f*_*Ca*_	0.0058 ± 0.00015		Fitted, this study.
*f*_*Mg*_	3.25 ± 0.052		Fitted, this study.
λ*_*onCa*_*	0		Postulated, this study
λ*_*onMg*_*	0		Postulated, this study
*K_*O*00_*	10800		Zahradnik et al., 2005
*k*_*CO*_	0.0011 ± 2.09E-05	ms^–1^	Fitted, this study.
*k*_*OC*_	11.9 ± 0.23	ms^–1^	Fitted, this study.
λ*_*CO*_*	0.671 ± 0.0015		Fitted, this study.
*K*_*IMg*_	546 ± 54	μM	(*k*_*IoffMg*_/*k*_*IonMg*_)^1/2^
*k*_*IonMg*_	5.9E-07 ± 2.6E-07	μM^–2^ ms^–1^	Fitted, this study.
*k*_*IoffMg*_	0.176 ± 0.084	ms^–1^	Fitted, this study.

*K_ACa_ – k_AoffCa_/k_AonCa_; Ca^2+^ dissociation constant of the RyR activation site in the closed state C00.*

*k_AonCa_ – Ca^2+^ binding rate constant of the RyR activation site in the closed state C00.*

*k_AoffCa_ – Ca^2+^ unbinding rate constant of the RyR activation site in the closed state C00.*

*K_AMg_ – k_AoffMg_/k_AonMg_; Mg^2+^ dissociation constant of the RyR activation site in the closed state C00.*

*k_AonMg_ – Mg^2+^ binding rate constant of the RyR activation site in the closed state C00.*

*k_AoffMg_ – Mg^2+^ unbinding rate constant of the RyR activation site in the closed state C00.*

*f_Ca_ – allosteric factor coupling Ca^2+^ binding to channel opening.*

*f_Mg_ – allosteric factor coupling Mg^2+^ binding to channel opening.*

*λ_onCa_ – partition contribution due to allosteric coupling on k_AonCa_.*

*λ_onMg_ – partition contribution due to allosteric coupling on k_AonMg_.*

*K_O00_ – k_CO_/k_OC_; dissociation constant of the ion-free open state O00.*

*k_CO_ – rate constant of the C00 → O00 transition.*

*k_OC_ – rate constant of the O00 → C00 transition.*

*λ_CO_ – partition contribution due to allosteric coupling on k_CO_.*

*K_IMg_ – (k_IoffMg_/k_IonMg_)^1/2^; dissociation constant of Mg^2+^ ions at the RyR inhibitory site.*

*k_IonMg_ – Mg^2+^ binding rate constant of the RyR inhibition site.*

*k_IoffMg_ – Mg^2+^ unbinding rate constant of the RyR inhibition site.*

*The value of k_AonMg_ was set to 0.01 × k_AonCa_, based on the generally accepted theory according to which the rate-limiting factor in ion binding to a ligand is the exchange rate of a water molecule in the hydration complex of an ion. The water exchange rate of Mg^2+^ is two orders of magnitude slower than that of the Ca^2+^ ion (Eigen and Wilkins, 1965).*

*Values of λ_onCa_ and λ_onMg_ were set to zero since Ca^2+^ binding to the ion-free closed state C00 is diffusion-limited (Zahradnikova et al., 1999). This means that the binding of Ca^2+^ and Mg^2+^ at the activation site of one monomer does not affect the on-rate constants but only the off-rate constants of these ligands binding to other monomers (see Supplementary Table 1).*

### Kinetic Equations of the Ryanodine Receptor Gating Model

The RyR gating model was represented by a transition rate matrix **Q** with *n* states defined as follows:


(1) $$Q_{i\,j}=\left\{ \begin{array}{c} q_{i\,j},i\neq j\\ -\sum_{j}q_{i\,j},i=j \end{array}\right.,$$

where *q*_*ij*_ is the transition rate between states *i* and *j*. The transition rates between RyR states are given in Supplementary Table 1 as functions of model parameters. The Q-matrix was used to calculate the mean open times and activation times of the model (Colquhoun and Hawkes, 1995). The mean open and closed times, *t*_*O*_ and *t*_*C*_, were calculated from the multi-exponential distributions of open and closed times (Colquhoun and Hawkes, 1995):


(2) $$t_{O}=\sum_{i=1}^{n_{0}}a_{i}\,\tau_{i},t_{C}=\sum_{j=1}^{n_{C}}a_{j}\,\tau_{j},$$

where *n*_*O*_ and *n*_*C*_ are the numbers of open and closed states and *a*_*i*_, *a*_*j*_ and *τ_*i*_*, *τ_*j*_* are the areas and the time constants of individual exponentials. To calculate *a*_*i*_ and *τ_*i*_*, the Q-matrix was partitioned into blocks:


(3) $$\text{Q}=\left[\begin{array}{cc} {\text{Q}}_{C\,C} & {\text{Q}}_{C\,O}\\ {\text{Q}}_{O\,C} & {\text{Q}}_{O\,O} \end{array}\right],$$

where C and O correspond to closed and open states, respectively; *τ_*i*_* = 1/λ*_*i*_*, where λ*_*i*_* is the *i^th^* eigenvalue of –***Q****_*OO*_*; and *a*_*i*_ = – τ_*i*_ **Φ***_0_* ***A****_*i*_* ***Q****_*OO*_* ***u****_*O*_*, where **Φ***_0_* is the initial vector, ***A****_*i*_* is the spectral matrix corresponding to eigenvalue λ*_*i*_*, ***u****_*O*_* is a column vector of ones with length equal to the number of open states. The values of *a*_*j*_ and *τ_*j*_* were calculated analogously using the submatrix ***Q****_*CC*_*.

The time courses of open probability *P*_*O*_ in response to a step change of [Ca^2+^] from the basal concentration [Ca^2+^]*_*b*_* to the end concentration [Ca^2+^]*_*e*_* were calculated from the system of Kolmogorov equations:


(4) $$\frac{d\,\text{P}\,\left(t\right)}{d\,t}=\text{P}\,\left(t\right)\,\text{Q}\,\left({\left[{\text{Ca}}^{2+}\right]}_{e}\right),$$

where***P*** (*t*) is the vector of probabilities of individual RyR states, as the sum of the time-dependent probabilities of all open states. The initial vector **P**(0) was calculated from the Q-matrix at [Ca^2+^]*_*b*_* as the solution of the system of equations (Colquhoun and Hawkes, 1995):


(5) $$\text{P}\,\left(0\right)\,\text{Q}\,\left({\left[{\text{Ca}}^{2+}\right]}_{b}\right)=0,$$

where **0** is a zero-vector with a length equal to the number of states. The activation time was defined as the time when open probability reaches 63.2%, i.e., (1– 1/*e*)-th part of the interval between open probabilities at [Ca^2+^]*_*b*_* and [Ca^2+^]*_*e*_*.

### Calcium Buffers, Diffusion, Ryanodine Receptor State Transitions, and Fluorescence

The description of Ca^2+^ diffusion in the CRS assumed open RyRs as a source of constant single-channel calcium current *i*_*Ca*_, flowing into calcium buffers that do not saturate (Naraghi and Neher, 1997). It also assumed instantaneous formation of free [Ca^2+^] gradients upon opening or closing RyR channels. The components and parameters of the calcium buffers in the CRS model (Table 2) correspond to published data and should represent the minimal requirements for proper RyR function under near-physiological conditions (Table 1; section “Kinetic Parameters of the Ryanodine Receptor Gating Model”). Concentrations of divalent ions binding to RyRs were calculated using constant 1 mM free [Mg^2+^] (Table 2), and basal 100 nM free [Ca^2+^] that was changed by calcium release. The released calcium equilibrated with Mg-unbound ATP, calmodulin, and the fluorescent dye Fluo-4 (Table 2). New Ca^2+^ concentration profiles were recalculated for each change in the RyR open state (see below).

**TABLE 2:** Calcium buffers used for calculation of Ca^2+^ concentrations and diffusion.

Components of the Ca buffer	*k*_*on,Ca*_ (μM^–1^ ms^–1^)	*k*_*off,Ca*_ (ms^–1^)	Concentration (μM)	*D* (μm^–2^ ms^–1^)	References
ATP (apparent total)	0.225	45	400*	0.14	Valent et al., 2007
Fluo-4	0.1	0.11	50	0.042	Hake et al., 2012
Calmodulin	0.023	0.238	24	0.025	
Troponin (cytosol only)	0.039	0.02	70	0	
Sarcolemma (cytosol only)	0.115	1	1124	0	Zahradnikova et al., 2007
SR membrane (cytosol only)	0.115	0.1	47	0	

*Basal free [Ca^2+^] was set to 100 nM.*

*The apparent total ATP represents the ATP concentration used to calculate free Ca^2+^ during calcium release. This value was calculated for a total ATP of 5 mM, total Mg of 5.6 mM, free [Mg^2+^] of 1 mM, and K_d,MgATP_ of 87 μM (Valent et al., 2007). We used a constant apparent total ATP to simplify calculations of [Ca^2+^] during calcium release, since the free Mg concentration stayed constant as well, and the released Ca^2+^ ions equilibrated effectively only with the apparent total ATP. The “cytosol only” components were used for calculation of the fluorescence signal in the cell cytosol volume.

The above assumptions allowed the use of the Naraghi-Neher approximation for calculations of free calcium concentrations during Ca flux in the cytosolic half-space delimited by the plane of the CRS (Naraghi and Neher, 1997) and the event-based Gillespie algorithm (Gillespie, 1977) for simulations. Briefly, each CRE was started by an opening of the selected RyR. The starting open state for the selected RyR was chosen from all possible open states based on their conditional probabilities *P*(*i* | channel is open) at basal concentrations of free Ca^2+^ (100 nM) and free Mg^2+^ (1 mM), i.e., probabilities of the states given that the channel is open:


(6) $$P\,\left(i|\mathrm{channel}\,\mathrm{is}\,\mathrm{open}\right)=\frac{P_{i}\,S_{i}}{\sum_{i=1}^{n}P_{i}\,S_{i}},$$

where *S*_*i*_ = 1 if the state *i* corresponds to an open state and 0 otherwise. The initial state of the closed channels was chosen based on the calculated probabilities from Eq. 6 but using (1 – *S*_*i*_) instead of *S*_*i*_. Calcium influx upon the first channel opening changed the calcium distribution in the CRS and, correspondingly, the Q-matrix of individual RyRs. The index of the RyR that would change its state was sampled from the distribution of probabilities of state transitions of individual RyRs, which was for the *j^th^* RyR equal to the ratio of its transition rate of leaving the microstate *M(j*), equal to –*Q*_*M*(_*_*j*_*_)_*_*M*_*_(_*_*j*_*_)_, to the sum of leaving rates over all the RyRs, $-\sum_{j=1}^{n}$ *Q*_*M*(_*_*j*_*_)_*_*M*_*_(_*_*j*_*_)_. The time of the next transition was sampled from the exponential distribution of waiting times with the rate $-\sum_{j=1}^{n}$ *Q*_*M*(_*_*j*_*_)_*_*M*_*_(_*_*j*_*_)_. If the transition changed the macrostate of the channel, i.e., opened a closed channel or closed an open channel, the transition would lead to a new distribution of [Ca^2+^] in the CRS and a new corresponding set of RyR transition rate constants. The algorithm was repeated until the end of the simulation. From each RyR in the CRS, a dataset of 500 CREs was collected at each set of tested parameters.

The fluorescence signals of CREs were calculated from the time course of calcium release flux (*N*_*O*_(*t*) ⋅ *i*_*Ca*_) as if emanating symmetrically from a virtual spherical source of 100 nm radius and binding to cytosolic buffers, including the calcium selective fluorophore Fluo-4 and the non-diffusible buffers troponin, sarcolemma, and SR membrane (Table 2), in a sphere of 10 μm radius. The intensity of fluorescence is directly proportional to the concentration of calcium-bound Fluo-4 [Ca^2+^-Fluo4], thus the change of fluorescence was reported as Δ*F*/*F*_0_. The calculated spatial profile of [Ca^2+^-Fluo4] was convolved with a Gaussian point spread function with FWHM = 400, 400, and 800 nm for the X, Y, and Z coordinate, respectively, to approximate the image observed by fluorescent microscopy (Smith et al., 1998; Iaparov et al., 2021).

### Parameter Space of the Simulations

To study how changes in Mg-RyR interactions affect the characteristics of CREs, the parameters of Mg^2+^ binding and unbinding were varied in a wide range relative to the reference values (Table 1). All values of Mg-binding parameters used in simulations are in Supplementary Table 2. The rate constants of the activation site were varied in the range [0.000596, 0.0271] μM^–1^ms^–1^ (*k*_*AonMg*_, 9 unique values) and [0.055, 2.5] ms^–1^ (*k*_*AoffMg*_, 9 unique values), resulting in 81 combinations that had *K*_*AMg*_ in the range [2.03, 4194] μM. The allosteric coefficient of Mg^2+^ at the RyR activation site was varied from 0.5 to 500, i.e., from 0.15× to 154× the reference value (Table 1) in 17 steps. The rate constants of the inhibition site were varied in the range between [1.84, 16.75] ⋅ 10^–7^ μM^–2^ms^–1^ (*k*_*IonMg*_, 9 unique values) and between [0.055, 0.5] ms^–1^ (*k*_*IoffMg*_, 9 unique values), resulting in 81 combinations that had *K*_*IMg*_ in the range [181.23, 1647.56] μM.

### Calcium Release Site and Calcium Release Events

Calcium release sites containing 20 RyRs with the geometric arrangement corresponding to a typical RyR cluster in cardiac dyads (Figure 1) were generated according to Jayasinghe et al. (2018). In brief, the first channel was placed on the coordinates (0, 0). The placement of further channels was defined by the distance from the preceding channel and the direction angle. The distance was sampled from a normal distribution with a mean of 40.1 nm and a standard deviation of 7.4 nm with a cutoff at 29 nm. The angle was sampled from the uniform distribution (0, 2π). To avoid overlap, a distance between RyRs of less than 29 nm was not allowed.

For quantitative characterization of the geometrical arrangement of RyRs, we used the concept of vicinity (Iaparov et al., 2021), an extension of the concept of adjacency (Walker et al., 2015) to arbitrary RyR-RyR distances. The RyR vicinities *v*_*i*_ and the group vicinity *v* were determined according to Iaparov et al. (2021) as:


(7) $$v_{i}=\frac{\sum_{j,j\neq i}v_{i\,j}}{N_{R\,y\,R}-1}=\frac{\sqrt{N_{R\,y\,R}}\,\sum_{j,j\neq i}C_{i\,j}}{N_{R\,y\,R}-1}\,\text{and}\,v=\frac{\sum_{i}v_{i}}{N_{R\,y\,R}},$$

where *N*_*RyR*_ is the number of RyRs in the CRS, and *C*_*ij*_ is the reciprocal value of the distance between the *i^th^* and the *j^th^* RyR (Iaparov et al., 2021). Out of 100 generated release sites, the one that showed the highest range of RyR vicinities was selected for simulations (Figure 1). The group vicinity of the CRS used in this study was *v* = 0.058 and the RyR vicinities spanned the range 0.034 - 0.072.

The simulation of a calcium release event was started by the opening of one of the RyRs and proceeded for 200 ms. Individual records of CREs were characterized by the number of RyRs open at the peak ($N_{O}^{p\,e\,a\,k}$), the time-to-peak (TTP), the time-to-end (TTE) of RyR activity, and the molar amount of Ca^2+^ released during the event (*n*_*Ca*_). Calcium release events were classified as quarks, blips, and sparks as previously described (Iaparov et al., 2021). Events consisting of one channel opening were defined as quarks. Blips and sparks were discerned by their $N_{O}^{p\,e\,a\,k}$ using the nadir of the amplitude histogram of CREs obtained with the reference set of CRS parameters. The nadir value for the reference model, determined by using the method of K-Means (MacQueen, 1967) implemented in scikit-learn (Pedregosa et al., 2011), was 6.2, thus CREs with 6 or fewer simultaneously open RyRs were classified as blips, and CREs with 7 and more simultaneously open RyRs were classified as sparks. The data were averaged for each dataset of 500 CREs that occurred in response to activation of one RyR under identical conditions.

### Coupling Strength

To understand the effect of magnesium more deeply, the quantitative relationship between the characteristics of CREs and determinants of CRSs, such as RyR placement, calcium current, and Mg^2+^ binding would be instrumental. Previously (Iaparov et al., 2021), we have introduced the coupling strength φ as a descriptor of CREs that weights the vicinity of RyRs and the calcium current through individual RyRs:


(8) $$\varphi_{i}=v_{i}\,{\left(i_{C\,a}\right)}^{\alpha },$$

where the index *i* enumerates individual RyRs of the CRS and the exponent α weights the contribution of *i*_*Ca*_ relative to vicinity *v*_*i*_. It should be noted that *v*_*i*_ values are calculated from RyR coordinates in the CRS (section “Calcium Release Site and Calcium Release Events”), *i*_*Ca*_ is defined in the model, so the respective coupling strength can be directly evaluated for each CRS model if the weight factor α can be found. As shown in Iaparov et al. (2021), the relation between either *v*_*i*_ or *i*_*Ca*_ and the measured characteristics of the corresponding simulated calcium release events was suboptimal, which could be misinterpreted as if the CREs were not fully controlled by the determinants of CRS, at odds with current understanding of local calcium release. Optimization of the weight factor allowed to find coupling strength values that correlated with the measured CRE characteristics according to Hill function for the whole parameter range of inspected CRS determinants (Iaparov et al., 2021).

To account for the effect of Mg-RyR binding parameters on the coupling strength among RyRs, we included terms for allosteric coupling (1/*f*_*Mg*_), activation (*k*_*AoffMg*_, *k*_*AonMg*_), and inhibition (*k_*IoffMg*_, k_*IonMg*_*) and defined the effective coupling strength as


(9a) $$\varphi_{i}^{e\,f\,f\,F}=v_{i}\,{\left(i_{C\,a}\right)}^{\alpha }\,{\left(\frac{1}{f_{M\,g}}\right)}^{\beta },$$

(9b) $$\varphi_{i}^{e\,f\,f\,A}=v_{i}\,{\left(i_{C\,a}\right)}^{\alpha }\,\frac{{\left(k_{A\,o\,f\,f\,M\,g}\right)}^{\gamma_{o\,f\,f}}}{{\left(k_{A\,o\,n\,M\,g}\right)}^{\gamma_{o\,n}}},$$

(9c) $$\varphi_{i}^{e\,f\,f\,I}=v_{i}\,{\left(i_{C\,a}\right)}^{\alpha }\,\frac{{\left(k_{I\,o\,f\,f\,M\,g}\right)}^{\delta_{o\,f\,f}}}{{\left(k_{I\,o\,n\,M\,g}\right)}^{\delta_{o\,n}}},$$

where the exponents β, γ*_*off*_*, γ*_*on*_*, δ*_*off*_*, and δ*_*on*_* are the weight factors that determine the contribution of the respective RyR kinetic parameters to CRS activity relative to the contribution of RyR vicinity, in analogy to the weight factor α of *i*_*Ca*_. According to the above formulae, the effective coupling strength between RyRs increases with increasing parameters that promote activation of other RyRs by a RyR opening, i.e., the vicinity, calcium current, and off-rate constants of Mg^2+^ binding. The effective coupling strength decreases with increasing parameters that hinder activation of other RyRs by a RyR opening, i.e., the allosteric factor and on-rate constants of Mg^2+^ binding. However, since the reference values of 1/*f*_*Mg*_, *k*_*AoffMg*_/*k*_*AonMg*_, and *k_*IoffMg*_/k_*IonMg*_* differed from each other, the values of coupling strengths $\varphi_{i}^{e\,f\,f\,F}$, $\varphi_{i}^{e\,f\,f\,A}$, and $\varphi_{i}^{e\,f\,f\,I}$ differed numerically from each other as well despite describing the same RyR model. To aid the comparison between the effect of Mg^2+^-binding parameters at the activation and the inhibition site, we normalized Mg^2+^-dependent parameters to the respective parameters of the reference RyR model (reported in Table 1), resulting in the effective coupling strength in the form:


(10a) $$\varphi_{i}^{e\,f\,f\,F}=v_{i}\,{\left(i_{C\,a}\right)}^{\alpha }\,{\left(\frac{1}{f_{M\,g}^{r\,e\,l}}\right)}^{\beta },$$

(10b) $$\varphi_{i}^{e\,f\,f\,A}=v_{i}\,{\left(i_{C\,a}\right)}^{\alpha }\,\frac{{\left(k_{A\,o\,f\,f\,M\,g}^{r\,e\,l}\right)}^{\gamma_{o\,f\,f}}}{{\left(k_{A\,o\,n\,M\,g}^{r\,e\,l}\right)}^{\gamma_{o\,n}}},$$

(10c) $$\varphi_{i}^{e\,f\,f\,I}=v_{i}\,{\left(i_{C\,a}\right)}^{\alpha }\,\frac{{\left(k_{I\,o\,f\,f\,M\,g}^{r\,e\,l}\right)}^{\delta_{o\,f\,f}}}{{\left(k_{I\,o\,n\,M\,g}^{r\,e\,l}\right)}^{\delta_{o\,n}}},$$

where the superscript *rel* stays for a value relative to the reference value, and the remaining parameters have been already defined.

### Determination of Weight Factors of the Effective Coupling Strength

Finding the effective coupling strength function (Eqs. 8-10) requires evaluation of the weight factors of individual components, namely, the RyR vicinity, single-channel current, and kinetic parameters of the activation and inhibition processes. The values of weight factors can be found by optimizing the effective coupling strength function to the distribution of measured CRE characteristics (${\bar{N}}_{O}^{p\,e\,a\,k}$, *F*_*q*_, and *F*_*s*_). Iaparov et al. (2021) determined the values of weight factors by using the Hill function to optimize the relationships between coupling strength (Eq. 8) on the one hand, and the relative amplitudes of CREs (*A*_*rel*_) and frequency of quarks and sparks (*F*_*q*_ and *F*_*s*_) on the other hand by a proper weight factor. In the present case, however, it would not be appropriate to optimize the functional relationships between the effective coupling strength and CRE characteristics $N_{O}^{p\,e\,a\,k}$, *F*_*q*_, and *F*_*s*_ using the Hill function, since the Mg-binding parameters do not characterize the state of the CRS but kinetic processes in the RyR molecule. Defining an *a priori* relational function could lead to a mutual dependence of the fitted parameters and a potential bias in weight factor estimates. To avoid this, we approached this problem using information theory as follows:

For a random variable (*X*) equal to the effective coupling strength, there is a dependent variable (Y) representing the CRE characteristics. The information content of a random variable *X* is quantified by its entropy *H*(*X*) (Shannon, 1948) that is calculated based on the probability density function *P(X*) of the variable. For a discrete variable,


(11) $$H\,\left(X\right)=-\sum_{i=1}^{n}P\,\left(x_{i}\right)\cdot \log P\,\left(x_{i}\right),$$

where *n* is the number of possible values of X. The entropy *H*(*X*) defines the uncertainty of the random variable. A large value of *H* indicates a large number of states with low probabilities and the measurement of such a variable provides a large amount of information, while *H* = *0* means a deterministic outcome and the measurement of such a variable will not provide any information because its value is exactly known.

We have calculated the mutual information between the effective coupling strength and the CRE characteristics. Mutual information (MI) between random variables *X* and *Y*, *I*(*X, Y*) is the amount of information obtained when *X* is known and vice versa (Shannon, 1948), defined through entropies:


(12) I⁢(X,Y)=H⁢(X)+H⁢(Y)-H⁢(X,Y),

where $H\,\left(X,Y\right)=-\sum_{i=1}^{n}\sum_{j=1}^{m}P\,\left(x_{i},y_{i}\right)\cdot \log P\,\left(x_{i},y_{i}\right)$ is called the joint entropy. Mutual information is a non-negative number being zero when *X* and *Y* are independent variables. This definition indicates that when the maximal MI between the effective coupling strength and the CRE characteristics is reached, the effective coupling strength provides the most certain prediction for CRE characteristics.

We maximized the average MI estimated between effective coupling strengths and CRE characteristics ${\bar{N}}_{O}^{p\,e\,a\,k}$, *F*_*q*_, and *F*_*s*_ (9 in total). MI was calculated using a non-parametric method based on entropy estimation from k-nearest neighbor distances (Kraskov et al., 2004) implemented in scikit-learn (Pedregosa et al., 2011). Average MI was maximized using the differential evolution implemented in scipy (Virtanen et al., 2020). The estimated maximal average MI was 2.6 bits. The parameters with their estimated standard deviations are summarized in Table 3 of Results. The standard deviations were calculated using the bootstrap method from 100 bootstrap samples because of the high computational complexity of the MI estimation. For each bootstrap sample, new weights were found using the Nelder-Mead method implemented in scipy (Virtanen et al., 2020).

**TABLE 3:** Parameters and weight factors of the effective coupling strength.

Parameter	Weight factor		Fold change of $\varphi_{i}^{e\,f\,f}$	
		
	Name	Value	Reference value × 0.5	Reference value × 2
*i*_*Ca*_	α	0.934 ± 0.016	0.52	1.91
*f*_*Mg*_	β	0.146 ± 0.004	1.11	0.9
*k*_*Aoff*_	γ*_*off*_*	1.41 ± 0.01	0.38	2.66
*k*_*Aon*_	γ*_*on*_*	1.014 ± 0.0002	2.02	0.5
*k*_*Ioff*_	δ*_*off*_*	0.663 ± 0.014	0.63	1.58
*k*_*Ion*_	δ*_*on*_*	1.444 ± 0.022	2.72	0.37

*Data are given as the mean and SE of the fit.*

### Frequency of Calcium Release Events

The frequency of all spontaneous RyR openings in a group of *N*_*RyR*_ channels was calculated according to Kunze et al. (1985):


(13) $$f=N_{R\,y\,R}/\left({\left(t_{O}\right)}_{b}+{\left(t_{C}\right)}_{b}\right),$$

where (*t*_*O*_)*_*b*_* and (*t*_*C*_)*_*b*_* are the values of the RyR open and closed time at basal [Ca^2+^].

## Results

To study the role of Mg^2+^ ions in the activation of calcium release we turned to *in silico* experiments to overcome the complexities and uncertainties related to real experiments. We used the model of a calcium release site (CRS) based on a quantitative description of RyR gating that accounted for Ca^2+^ and Mg^2+^ binding kinetics, RyR distribution in a typical CRS, and the cytosolic Ca^2+^ buffer near RyRs and around the CRS. The reference parameter set of RyR gating was obtained by fitting the available experimental data of RyR open probability, open time, and activation time at a range of cytosolic concentrations of free Ca^2+^ and free Mg^2+^ that encompassed the physiological values (section “Kinetic Parameters of the Ryanodine Receptor Gating Model”). Then we analyzed the characteristics of simulated calcium release events generated at the physiological free [Mg^2+^] concentration (1 mM) with the reference RyR model (section “Characteristics of Simulated Calcium Release Events for the Calcium Release Site With the Monod-Wyman-Changeux-Ca/Mg Ryanodine Receptor Model”). The consequences of variation in parameters related to Mg^2+^ binding on the activity of RyR channels and the ensuing CREs were determined in sections “Effect of Mg-Binding Parameters on Calcium Dependence of Ryanodine Receptor Activation” and “Mg-Binding Parameters Affect Calcium Release Events.” Finally, we solved the relationship between RyR activity and the characteristics of CREs on the grounds of the effective coupling strength that, in addition to RyR vicinity and calcium current, accounts also for magnesium binding to RyR, and determined the effect of Mg-binding parameters on the characteristics of CREs (sections “Effective Coupling Strength Tallies Ryanodine Receptor Vicinity, Calcium Current, and Mg-Binding Parameters” and “The Frequency of Spontaneous Calcium Release Events Depends on the Ryanodine Receptor Closed Time of at Basal [Ca2+]”).

### Construction and Validation of the Ryanodine Receptor Gating Model

We developed a new model of RyR gating based on RyR single-channel studies of Ca^2+^ activation and Mg^2+^ inhibition (Zahradnikova et al., 1999, 2003; Tencerova et al., 2012; Cannell et al., 2013) and on the allosteric homotetrameric gating RyR model (Zahradnik et al., 2005; Zahradnikova et al., 2010). To reduce computational costs we omitted the long-lived L- and I-mode states of the original aHTG RyR gating model since their frequency of occurrence was much less than once per 200 ms (the duration of simulations). The gating scheme of the resulting Monod-Wyman-Changeux type, Ca^2+^- and Mg^2+^-binding (MWC-Ca/Mg) RyR model has 60 states (Figure 2). The kinetics was characterized by on/off rate constants of competitive binding of Ca^2+^ and Mg^2+^ to the RyR activation site allosterically coupled to channel opening, and on/off rate constants of Mg^2+^ binding to the RyR inhibition site that renders the channel non-conductive upon Mg^2+^ binding (see Supplementary Data).

The steady-state open probability of the MWC-Ca/Mg RyR gating model was described by Eq. 14 derived in the Supplementary Data:


(14) $$P_{O}\,\left(\left[C\,a^{2+}\right],\left[M\,g^{2+}\right]\right)=\frac{{\left(K_{I\,M\,g}\right)}^{2}}{{\left(K_{I\,M\,g}\right)}^{2}+{\left[M\,g^{2+}\right]}^{2}}.\frac{\begin{array}{c} {\left(\left[{\mathrm{Ca}}^{2+}\right]+K_{\mathrm{ACa},\cdot }\,f_{\mathrm{Ca}}\,\left(1+\frac{\left[{\mathrm{Mg}}^{2+}\right]}{K_{\mathrm{AMg}}\cdot f_{\mathrm{Mg}}}\right)\right)}^{4} \end{array}}{\begin{array}{c} {\left(\left[{\mathrm{Ca}}^{2+}\right]+K_{\mathrm{ACa},\cdot }\,f_{\mathrm{Ca}}\,\left(1+\frac{\left[{\mathrm{Mg}}^{2+}\right]}{K_{\mathrm{AMg},\cdot }\,f_{\mathrm{Mg}}}\right)\right)}^{4}+K_{\mathrm{O00},\cdot }\,{\left(f_{\mathrm{Ca}}\right)}^{4}\,{\left(\mathrm{Ca}+K_{\mathrm{ACa}}\,\left(1+\frac{\left[{\mathrm{Mg}}^{2+}\right]}{K_{\mathrm{AMg}}}\right)\right)}^{4} \end{array}}.$$

**FIGURE 3:**
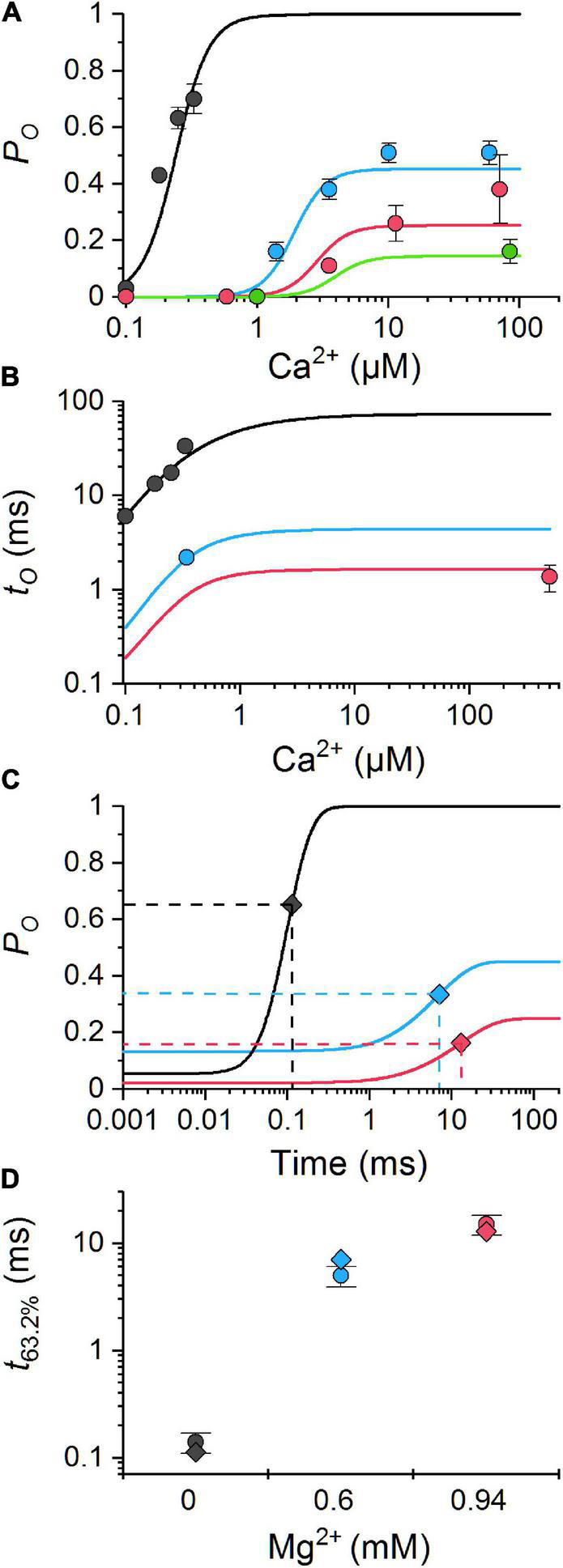
Comparison of the MWC-Ca/Mg RyR model predictions with experimental data. The model predictions (lines) were calculated using Eqs. 14 **(A)**, 2 **(B)**, and 4 **(C)** and the reference set of parameter values (Table 1) for the same ion conditions as the experimental data (points). **(A)** The calcium dependence of open probability for 0 mM Mg^2+^ and 0.5 mM total ATP (Tencerova et al., 2012) (black), and for 0.6, 0.94, and 1.33 mM free Mg^2+^ (blue, red, and green, respectively) and 3 mM total ATP (Zahradnikova et al., 2003). **(B)** The calcium dependence of mean open time for 0 mM total Mg^2+^ and 0.5 mM ATP (Tencerova et al., 2012) (black), for 0.6 mM free Mg^2+^, 3 mM total ATP (Zahradnikova et al., 2003) (blue), and for 1 mM free Mg^2+^ and 2 mM total ATP (Cannell et al., 2013) (red). **(C)** The simulated time course of RyR responses to a step Ca^2+^ increase from 100 nM to 20 μM in the absence of Mg^2+^ (black), and from 1.5 to 10 μM Ca^2+^ in the presence of 0.6 and 0.94 mM free Mg^2+^ and 3 mM total ATP (blue and red lines, respectively). The 63.2% activation time is indicated by diamonds. **(D)** Comparison of the calculated (diamonds) and experimental (dots with error bars) 63.2% activation times obtained under conditions shown in panel **(C)**; black - (Zahradnikova et al., 1999), red and blue - (Zahradnikova et al., 2003).

The meaning of the model parameters in Eq. 14 is provided in Table 1 together with their values. The values were estimated by optimizing the global approximation of the experimental data shown in Figure 3 with the respective functions (Eqs. 2, 4, and 14). The optimized model predicts the Ca^2+^-dependences of the open probability, the open time, and the rate of activation by Ca^2+^ at different Mg^2+^ concentrations in a very good agreement with the available experimental data (Figure 3). We will refer to this set of optimized parameters as the reference RyR model or parameter set. Note that for the reference RyR model and the free [Mg^2+^] of 1 mM (red lines in Figure 3), the maximum steady-state open probability *P_*O*_*^max^** was 0.23 and the open time *t_*O*_*^max^** was 1.66 ms.

### Characteristics of Simulated Calcium Release Events for the Calcium Release Site With the Monod-Wyman-Changeux-Ca/Mg Ryanodine Receptor Model

In the CRE simulations, we used the approximation of constant single-channel calcium current during RyR openings, which allowed us to analyze the effects of Mg^2+^ binding kinetics on the activation of calcium release without the multifaceted effects brought about by the inclusion of SR depletion in models of the CRS.

**FIGURE 4:**
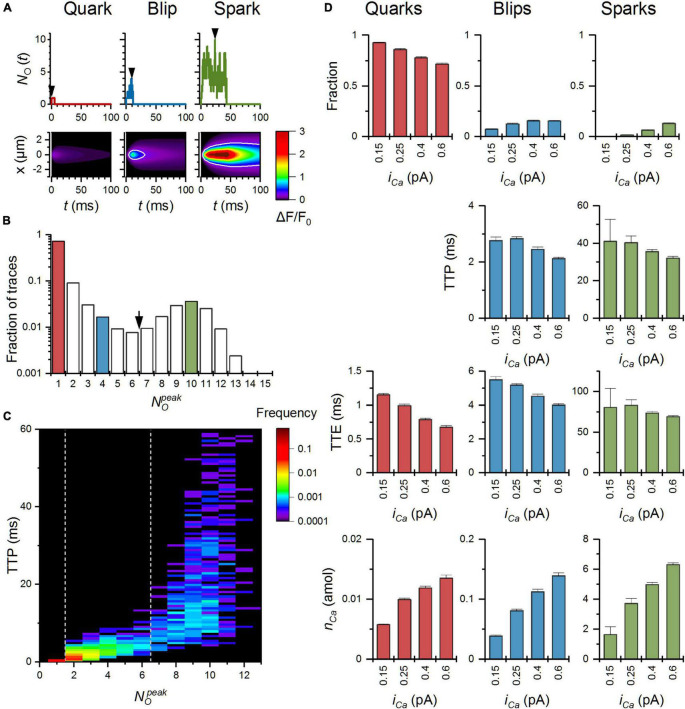
Characteristics of calcium release events (CREs) simulated with the MWC-Ca/Mg RyR model and the reference set of parameters. **(A)** Top panel – the time courses of simulated CREs representing a typical quark, blip, and spark for *i*_*Ca*_ = 0.6 pA; Bottom panel – the fluorescence calcium signals emulating laser line-scan images in a confocal microscope corresponding to CREs in panel **(A)**. **(B)** The distribution of the peak number of open RyRs, $N_{O}^{p\,e\,a\,k}$, in the simulated calcium release events. The red, blue, and green columns correspond to peaks of respective traces shown in panel **(A)**. **(C)** The surface plot of the time to peak vs. $N_{O}^{p\,e\,a\,k}$; dotted lines indicate the $N_{O}^{p\,e\,a\,k}$ values used for discrimination between quarks, blips, and sparks. **(D)** Relationships between *i*_*Ca*_ values and characteristics of simulated quarks, blips, and sparks; *n*_*Ca*_ – the amount of Ca^2+^ released *per* CRE.

Calcium release events simulated with the reference parameter set of the MWC-Ca/Mg RyR model (Figures 4A-C) consisted of three event types as observed previously for CRSs constructed with the two-state Ca/Mg RyR model of Iaparov et al. (2021). Although the amplitude histograms of both models differed substantially in the relative fractions of CRE types, the nadir of histograms at ∼6 and the most frequent $N_{O}^{p\,e\,a\,k}$ of sparks at ∼10 RyRs were the same in both models. The fraction of events with $N_{O}^{p\,e\,a\,k}$ ≤ 6 (quarks and blips) was much larger in the present model.

As shown in Figure 4D, the fraction of quarks decreased with the *i*_*Ca*_ value (93% at 0.15 pA and 72% at 0.6 pA). In comparison with CRSs based on the two-state RyR model of Iaparov et al. (2021), the decrease was much weaker. The fraction of blips increased with *i*_*Ca*_ (7% at 0.15 pA and 15% at 0.6 pA) on account of quarks, while in the two-state model it decreased with *i*_*Ca*_ since in the two-state model the fraction of sparks increased more steeply with *i*_*Ca*_ than in the MWC-Ca/Mg RyR model (0.06% of sparks at 0.15 pA and 13% of sparks at 0.6 pA in the present model). The time to peak of blips and sparks was curtailed at higher *i*_*Ca*_, similar to the two-state model. However, the time to peak of sparks decreased with *i*_*Ca*_ in the present model while it increased in the two-state model. The time to end of quarks and blips shortened with *i*_*Ca*_, as it did in the two-state model. In contrast to the two-state model, the time to end of sparks did not depend significantly on *i*_*Ca*_. The amount of calcium released by CREs increased proportionally with *i*_*Ca*_, less steeply than in the two-state model.

### Effect of Mg-Binding Parameters on Calcium Dependence of Ryanodine Receptor Activation

**FIGURE 5:**
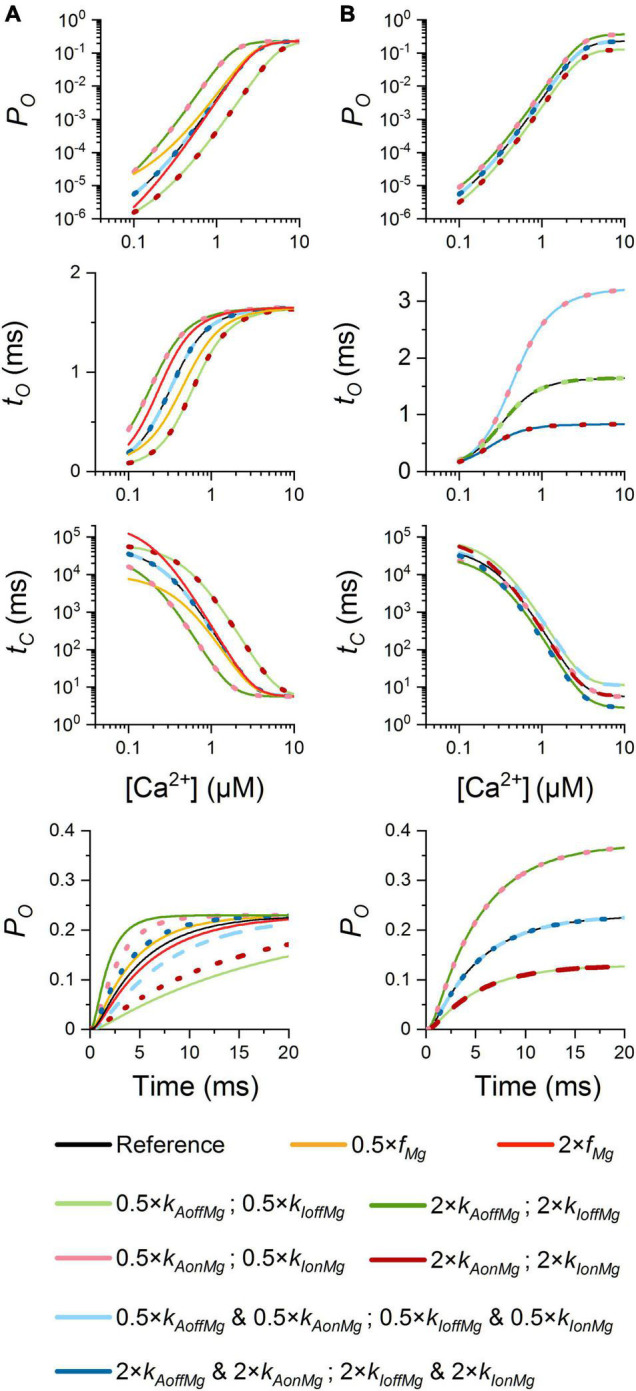
Simulated dependence of RyR single-channel characteristics on Mg^2+^ binding. *P*_*O*_([Ca^2+^]), *t*_*O*_([Ca^2+^]), *t*_*C*_([Ca^2+^]), and *P*_*O*_(*t*) were calculated with Eqs. 14, 2, 2, and 4, respectively. **(A)** Variation of Mg-binding to the RyR activation site. **(B)** Variation of Mg-binding to the RyR inhibitory site. Bottom panels: time courses of *P*_*O*_ evolution after a stepwise increase of [Ca^2+^] from 0.1 to 20 μM. Color-coding is described in the legend. The conditions that had the same effect are shown in solid, dashed, or dotted lines. Note the logarithmic scale of *P*_*O*_ and *t*_*C*_.

Before characterization of calcium release events in the CRS, we inspected the single-channel RyR behavior of the MWC-Ca/Mg RyR model for a range of Mg-binding parameters at the physiological cytosolic free Mg^2+^ concentration of 1 mM. The calculated dependences of RyR characteristics on [Ca^2+^] for the allosteric coefficient *f*_*Mg*_ at the RyR activation site and the on/off rate constants of Mg^2+^ at both the activation and the inhibition sites are summarized in Figure 5. These data confirm the validity of the RyR model and will be further used to analyze how the effect of a parameter change on RyR single-channel kinetics translates to its effect on calcium release events.

#### Mg-Binding Parameters of the Ryanodine Receptors Activation Site

As shown in Figure 5A, changes that facilitated Mg^2+^ binding to the RyR activation site, that is, a decrease of *K*_*AMg*_ by either an increase of *k*_*AonMg*_ (purple), or a decrease of *k*_*AoffMg*_ (light green), led to decreased calcium sensitivity (right shift) of the RyR open probability, open time, and closed time. At the basal calcium concentration, these changes decreased *P*_*O*_ and *t*_*O*_ but increased *t*_*C*_. Vice versa, changes that impeded Mg^2+^ binding [a lower *k*_*AonMg*_ (pink) or a higher *k*_*AoffMg*_ (dark green)] had the opposite effect. A change of *K*_*AMg*_ led to a change of the apparent *K*_*Ca*_ in inverse proportion. Changing both on- and off-rate constants by the same fraction, that is, without changing *K*_*AMg*_ (light and dark blue), did not affect the calcium dependence of *P*_*O*_, *t*_*O*,_ and *t*_*C*_.

Variation of the allosteric coefficient *f*_*Mg*_ relative to the reference value had a non-linear effect on the calcium dependence of RyR characteristics (orange and red; Figure 5A). It affected RyR open probability and closed time only at sub-micromolar [Ca^2+^] range (well below its apparent *K*_*Ca*_ of 3.02 μM at 1 mM Mg^2+^), while it shifted the calcium dependence of open time in this [Ca^2+^] range but had no effect at low and high [Ca^2+^] range. Notably, at the lowest calcium concentration, a larger *f*_*Mg*_ decreased *P*_*O*_ and substantially increased *t*_*C*_, while a smaller *f*_*Mg*_ increased *P*_*O*_ and decreased *t*_*C*_. Variation of *f*_*Mg*_ exerted no effect on the maximal *P*_*O*_, *t*_*O*_, or minimal *t*_*C*_, in agreement with Eqs. 2 and 14.

The rate of RyR activation by a step change of [Ca^2+^] was speeded up the most by an increased rate constant of Mg^2+^ unbinding from the RyR activation site, *k*_*AoffMg*_ (dark green), somewhat less by decreasing *k*_*AonMg*_ (pink), and even less when both rate constants were increased in proportion (dark blue). The RyR activation rate was slowed down the most by decreased *k*_*AoffMg*_ (light green) or increased *k*_*AonMg*_ (purple), but less when both on- and off-rate constants of Mg^2+^ binding decreased (light blue). Changes of *f*_*Mg*_ had negligible effects on the rate of RyR activation.

In other words, the changes of Mg-binding at the RyR activation site modify the calcium sensitivity and kinetics of RyR activation but do not influence the maximum mean open probability, the maximum mean open time, or the minimum closed time.

#### Mg-Binding Parameters of the Ryanodine Receptors Inhibition Site

By definition, changes of the on- and off-rate constants for Mg^2+^ binding at the RyR inhibition site do not affect the calcium sensitivity of RyR open probability (3.02 μM at 1 mM [Mg^2+^]), but they had a profound effect on the maximum open probability (at 1 mM [Mg^2+^]; Figure 5B). Increasing *k*_*IonMg*_ (purple) or decreasing *k*_*IoffMg*_ (light green) decreased *P*_*O*_, while increasing *k*_*IoffMg*_ (dark green) or decreasing *k*_*IonMg*_ (pink) increased *P*_*O*_. In effect, a change of *K*_*IMg*_ led to an almost proportional change of the maximum *P*_*O*_. Changing both *k*_*IonMg*_ and *k*_*IoffMg*_ proportionally (light and dark blue; no change of *K*_*IMg*_) did not affect *P*_*O*_ at any [Ca^2+^]. On the other hand, the values of *t*_*O*_ were insensitive to changes of *k*_*IoffMg*_. However, a change of *k*_*IonMg*_ or a proportional change of both *k*_*IonMg*_ and *k*_*IoffMg*_ affected the calcium sensitivity of *t*_*O*_ in the direction of the change but changed the maximum *t*_*O*_ in the opposite direction.

At the basal [Ca^2+^], the open time was not affected by rate constants of Mg^2+^ binding to the inhibition site. However, increasing *k*_*IonMg*_ or decreasing *k*_*IoffMg*_ increased the closed time, and decreasing *k*_*IonMg*_ or increasing *k*_*IoffMg*_ decreased it. Simultaneous equivalent changes in both *k*_*IonMg*_ and *k*_*IoffMg*_ did not affect *t*_*C*_. At high [Ca^2+^], change of *k*_*IoffMg*_ or both *k*_*IonMg*_ and *k*_*IoffMg*_ in one direction changed the mean closed time in the opposite direction; however, changes of *k*_*IonMg*_ in either direction did not change it. The parameters of Mg^2+^ binding to the inhibition site did not affect the rate constant of RyR activation, as expected.

Notably, the Mg-RyR interaction at the inhibitory site transpires through RyR open time when the rate of Mg^2+^ binding is modulated, and through RyR closed time when the rate of Mg^2+^ unbinding is modulated. The changes of open and closed time evoked by RyR-Mg^2+^ interaction at the inhibitory site are not dependent on Ca^2+^ concentration.

### Mg-Binding Parameters Affect Calcium Release Events

The relative occurrence of sparks steeply declined at lower *i*_*Ca*_ values (Figure 4D). Therefore, to assure an acceptable signal-to-noise ratio of the average sparks, we have used only the single-channel calcium current of 0.6 pA to analyze the effect of Mg-binding parameters on the time course of CREs.

**FIGURE 6:**
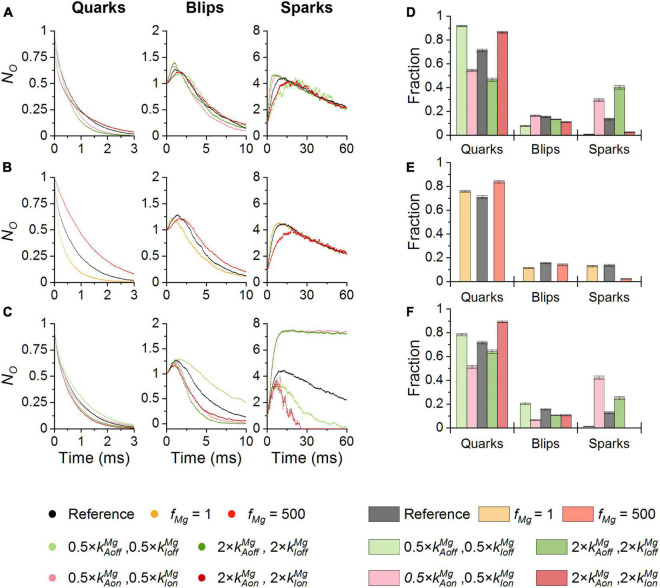
The effect of Mg-binding parameters on CREs. Simulations were performed at 100 nM Ca^2+^ and *i*_*Ca*_ of 0.6 pA. **(A,D)** The effect of Mg-binding parameters at the activation site on the time course **(A)** and the fraction **(D)** of CREs. **(B,E)** The effects of the allosteric coefficient *f*_*Mg*_ on the time course **(B)** and the fraction **(E)** of CREs. **(C,F)** The effect of Mg-binding parameters at the inhibition site on the time course **(C)** and the fraction **(F)** of CREs. Individual conditions are color-coded as indicated in the legend.

Changes of the on- and off-rate constants of Mg^2+^ binding to the RyR activation site (Figure 6A) had a small effect on the decay rate of quarks. At a low allosteric coefficient *f*_*Mg*_ (orange) the quarks were shorter, and at a high *f*_*Mg*_ (red) they were longer than at the reference *f*_*Mg*_ (Figure 6B). Changes of the on- and off-rate constants of Mg^2+^ binding to the RyR inhibition site (Figure 6C) did not affect the time course of quarks substantially.

The amplitude and time course of blips were practically unaffected by changes of Mg-binding parameters of the RyR activation site (Figure 6A) or *f*_*Mg*_ (Figure 6B). Blips were strongly prolonged when *k*_*IoffMg*_ was decreased (light green) but the remaining changes of Mg^2+^-binding parameters of the RyR inhibition site slightly curtailed them. Increased *k*_*IonMg*_ (purple) curtailed blips due to their rapid attrition, while decreased *k*_*IonMg*_ (pink) or increased *k*_*IoffMg*_ (dark green) due to the rapid formation of sparks (Figure 6C).

The amplitude of sparks was substantially affected by changes of Mg^2+^ binding to the RyR inhibition site (Figure 6C), while other parameters had only a marginal effect. Increasing *k*_*IonMg*_ (purple) or decreasing *k*_*IoffMg*_ (light green) decreased the peak number of open RyRs, while increasing *k*_*IoffMg*_ (dark green) or decreasing *k*_*IonMg*_ (pink) increased it. Spark activation was slowed down when Mg^2+^ binding to either the activation site or the inhibition site was facilitated by increased on-rates (purple; Figures 6A,C) or decreased off-rates (light green; Figures 6A,C), or by an increased *f*_*Mg*_ (red; Figure 6B). It was speeded up when Mg^2+^ binding to either the activation site or the inhibition site was impeded by decreased on-rates (pink; Figures 6A,C) or increased off-rates (dark green; Figures 6A,C). Termination of sparks was virtually unaffected by the parameters of Mg^2+^ binding to the RyR activation site (Figure 6A). Increased Mg^2+^ binding to the RyR inhibition site curtailed sparks while decreased Mg^2+^ binding strongly prolonged them in addition to increasing their amplitude (Figure 6C).

The relative occurrence of CRE types (Figures 6D-F) was sensitive to the value of Mg-binding parameters as well. At the RyR activation site, the rate constants of Mg^2+^ binding/unbinding affected the relative fractions of quarks and sparks reciprocally, while the fraction of blips changed only slightly. Increased *k*_*AonMg*_ (light purple) and decreased *k*_*AoffMg*_ (light green) increased the fraction of quarks and reduced the fraction of sparks. Decreased *k*_*AonMg*_ (pink) and increased *k*_*AoffMg*_ (green) had the opposite effect. The decreased *f*_*Mg*_ (orange) did not affect the fractions of CREs but the increased *f*_*Mg*_ (red) strongly reduced the fraction of sparks and increased the fraction of quarks. At the RyR inhibition site, the parameters of Mg^2+^ binding, i.e., *k*_*IonMg*_ and *k*_*IoffMg*_, affected the fractions of quarks and sparks similarly as *k*_*AonMg*_ and *k*_*AoffMg*_ did. While at the activation site the off-rate constant *k*_*AoffMg*_ had a stronger effect, at the inhibition site the on-rate constant *k*_*IonMg*_ had a stronger effect.

These findings can be summarized as follows: the interaction of Mg^2+^ with the RyR activation site predominantly affects the fraction of quarks, blips, and sparks and modulates the time to peak of sparks. On the other hand, the interaction of Mg^2+^ with the RyR inhibition site affects the recruitment of RyRs in sparks and thus modulates amplitudes of sparks, as well as the fractions of quarks, blips, and sparks.

### Effective Coupling Strength Tallies Ryanodine Receptor Vicinity, Calcium Current, and Mg-Binding Parameters

We have shown above how the Mg-binding kinetics of RyR impacts the calcium dependence of single-channel activity and also how it influences the characteristics of calcium release events. Here we addressed how the kinetics of Mg^2+^ binding to RyRs combines with fundamental determinants of the calcium release site - RyR vicinity and single-channel calcium current. In other words, we evaluated their relative importance in shaping CRS activity. For this, we maximized mutual information between $\varphi_{i}^{e\,f\,f}$ and CRE characteristics ($N_{O}^{p\,e\,a\,k}$, *F*_*q*_ and *F*_*s*_) evaluated for each RyR separately for all values of Mg-binding parameters (see Supplementary Table 2) and all values of *i*_*Ca*_, and found the optimal values of weight factors (Table 3).

Weight factors for the calcium current and the on-rate of Mg-binding to the activation site are close to 1, so their effect is similar to that of the RyR vicinity. Factors for the off-rate constant of the activation site and the on-rate constant of the inhibition site have a stronger effect than RyR vicinity. Factors for the allosteric coefficient of Mg^2+^ and the off-rate constant of the inhibition site have a weaker effect than RyR vicinity.

**FIGURE 7:**
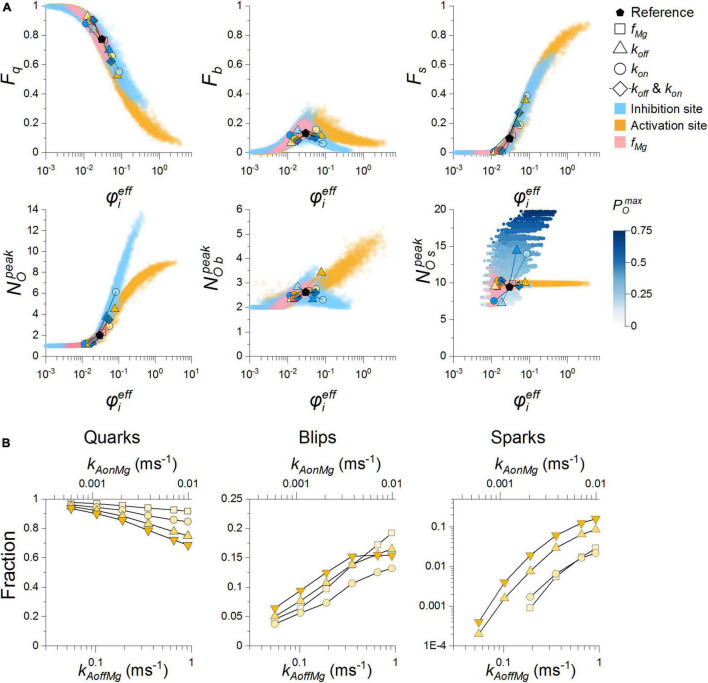
Calcium release event characteristics related to the effective coupling strength. **(A)** The whole dataset (14240 points per panel) for various Mg-binding parameters of the RyR activation site (orange and pink) and the RyR inhibition site (blue), which were obtained for all RyR vicinities and all single-channel calcium currents (not indicated). The dots are semitransparent to better visualize the data. The circles, triangles, and diamonds mark results for decreased (×0.5; lighter color) or increased (×2; darker color) Mg-binding parameter values (see legend), relative to the reference parameter values (black pentagons). The $P_{O}^{\max}$ colormap refers solely to the $N_{O\,S}^{p\,e\,a\,k}$ graph to indicate the relation of the $N_{O\,S}^{p\,e\,a\,k}$ increase to $P_{O}^{\max}$ increase when varying Mg-binding at the inhibitory site, not present in the case of blips. **(B)** The dependence of the fraction of quarks, blips, and sparks on the reciprocally changed rate constants *k*_*AonMg*_ (top axis) and *k*_*AoffMg*_ (bottom axis), so that the equilibrium constant *K*_*AMg*_ stayed at the reference value of 92 μM. Squares, circles, up triangles and down triangles denote values of *i*_*Ca*_ = 0.15, 0.25, 0.4, and 0.6 pA, respectively.

The CRE characteristics evaluated for all RyR models obtained by variation of the Mg-binding kinetics and *i*_*Ca*_ are displayed against their effective coupling strength values (Figure 7A). Although all data are consistently united, there were diverging trends between the data related to the activation site and the inhibition site. This divergence suggests that the similar effect on the fractional occurrence of quarks, blips, and sparks caused by Mg^2+^ binding to either the activation sites or the inhibition site is caused by different molecular mechanisms (see below).

A closer inspection of Figure 7A (see pairs of symbols representing changes of individual Mg-binding parameters) revealed that characteristics of calcium release events react sensitively and specifically in proportion to the respective changes of the effective coupling strength. Changes at the activation site that increased the calcium sensitivity of *P*_*O*_ and *t*_*O*_ and speeded up the time course of activation had a strong activating effect on CREs, and those that decreased the calcium sensitivity of *P*_*O*_ and *t*_*O*_ and slowed down the time course of activation had an inhibitory effect. Comparably strong were the effects at the inhibition site that increased maximum *P*_*O*_ and maximum *t*_*O*_, but did not change the calcium sensitivity of *P*_*O*_ and even decreased the calcium sensitivity of *t*_*O*_ and slowed down the time course of activation.

A concurrent increase of *k*_*IonMg*_ and *k*_*IoffMg*_, that is, an unchanged equilibrium constant *K*_*IMg*_, suppressed the fractional occurrence of sparks and promoted that of quarks and vice versa. These effects materialized at an unchanged *P*_*O*_ and activation rate. Thus, they could be only attributed to the change in RyR open and closed times. Similar effects materialized in the opposite direction for a concurrent change in *k*_*AonMg*_ and *k*_*AoffMg*,_ where a decrease of these parameters suppressed the fractional occurrence of sparks and promoted that of quarks (Figure 7B) but their increase had only a small effect. The effect of *k*_*AonMg*_ and *k*_*AoffMg*_ on CREs occurred in the absence of effects on *P*_*O*_, *t*_*O*_ and *t*_*C*_ and thus could be only attributed to the change of the time course of *P*_*O*_ activation by increased [Ca^2+^]. The changes of coupling strength and CRE properties evoked by changing both on- and off-rate constants can be attributed to the unequal weights of the on- and off-rates (Table 3). Biophysically they are due to the unequal effect of the on- and off-rates on the open and closed times and the activation rate constants (Figure 5).

The relationships between the effective coupling strength and the fractions of quarks, blips, and sparks upon changes in *K*_*IMg*_ evoked by changed on- or off-rate of Mg^2+^ binding to the inhibition site were of similar direction and extent as those evoked by changes of Mg^2+^ binding to the activation site. There were, however, pronounced changes in the amplitude characteristics of CREs caused by the dependence of maximum RyR open probability on *K*_*IMg*_. As a result, the dependence of the peak number of open RyRs in all events (Figure 7A, blue symbols) sharply increased with *φ_*eff*_*. In contrast to changes in Mg^2+^ binding to the RyR activation site, the amplitude of blips did not increase monotonously but showed a maximum at control values of *k*_*IonMg*_ and *k*_*IoffMg*_, and the amplitudes of sparks increased with *φ_*eff*_* due to the dependence between *φ_*eff*_* and *K*_*IMg*_.

### The Frequency of Spontaneous Calcium Release Events Depends on the Ryanodine Receptor Closed Time at Basal [Ca^2+^]

The last problem we addressed in this study was the impact of Mg-binding parameters of the RyR on the frequency of spontaneous occurrence of individual calcium release types at basal [Ca^2+^]. The frequency of all events at the CRS was directly proportional to *N*_*RyR*_ and inversely proportional to the sum of *t*_*C*_ and *t*_*O*_ (Eq. 13 in section “Methods”). Since at basal [Ca^2+^] *t*_*C*_ >> *t*_*O*_, this effect, summarized in Table 4 for the conditions examined in Figure 5, was chiefly due to changes in *t*_*C*_. The event frequency was changed approximately in proportion (*k*_*AoffMg*_, *k*_*AoffMg*_) or inverse proportion (*k*_*AonMg*_, *k_*AonMg*_, f_*Mg*_*) to the change of rate parameters. All parameters of Mg^2+^ binding affect the event frequency and the fraction of sparks in the same direction, and therefore they have a pronounced effect on the spontaneous spark frequency at the examined *i*_*Ca*_ of 0.6 pA. Since the fraction of blips is only weakly affected by Mg-binding parameters, the frequency of blips was changed only modestly.

**TABLE 4:** The effect of Mg-binding parameters on the frequency of occurrence of spontaneous calcium release events.

Frequencies	*f*_*O*_ (s^–1^)		*f*_*Q*_ (s^–1^)		*f*_*B*_ (s^–1^)		*f*_*S*_ (s^–1^)	
Reference values	0.57		0.409		0.089		0.072	
**Parameter**	**× *0.5***	**× *2.0***	**× *0.5***	**× *2.0***	**× *0.5***	**× *2.0***	**× *0.5***	**× *2.0***

*f*_*Mg*_	2.37	0.17	1.74	0.118	0.314	0.029	0.307	0.021
*k*_*AoffMg*_	0.36	1.23	0.33	0.573	0.028	0.163	0.002	0.494
*k*_*AonMg*_	1.23	0.36	0.668	0.311	0.199	0.040	0.363	0.009
*k*_*AoffMg*_ & *k*_*AonMg*_	0.57	0.57	0.453	0.358	0.083	0.092	0.034	0.120
*k*_*IoffMg*_	0.32	0.93	0.251	0.597	0.065	0.100	0.004	0.233
*k*_*IonMg*_	0.88	0.36	0.45	0.321	0.059	0.038	0.371	0.001
*k*_*IoffMg*_ & *k*_*IonMg*_	0.54	0.64	0.313	0.534	0.078	0.084	0.149	0.022

*f_O_, f_Q_, f_B_, f_S_ – the frequency of spontaneous RyR openings, quarks, blips, and sparks per CRS, respectively. CRS simulations were performed at 100 nM Ca^2+^ and i_Ca_ = 0.6 pA.*

## Discussion

The *in silico* model of the calcium release site was based on a novel model of RyR gating that incorporated Mg^2+^ binding kinetics (MWC-Ca/Mg RyR; Figure 2). Despite the complexity of the RyR model (60 states), the CRS model with 20 RyRs, written in C++ and Python, could be run fast on a generally available desktop computer, which allowed in-depth inspection of Mg^2+^ binding on the behavior of CRS.

### Mg^2+^ Ions and Ryanodine Receptor Sensitivity to Calcium

The parameter values of the MWC-Ca/Mg RyR model (Table 1) were determined from RyR single-channel characteristics measured in independent bilayer experiments under conditions that included physiological concentrations of ATP (approx. 5 mM total) and Mg^2+^ ions (1 mM free; Figure 3). The close approximation of the experimental data by simulations of the MWC-Ca/Mg RyR model validated both the computational model as well as the best-fit parameter values used as the reference set for simulations. Nevertheless, the fitted set of experimental data, which was obtained at a limited range of Ca^2+^ and Mg^2+^ concentrations, has not been verified by others. Thus, the estimated reference parameter values may differ from the true values despite their high statistical agreement with the data. Therefore, we varied the Mg-binding parameters of the RyR model around their reference values at a constant Mg^2+^ concentration and calculated the RyR single-channel characteristics for a full scale of cytosolic Ca^2+^ concentrations (Figure 5).

The tested RyR models showed a high sensitivity of RyR activation to parameters of Mg-binding. Increased Mg^2+^ affinity of the RyR activation site, achieved by variation of binding/unbinding rate constants, shifted the calcium dependence of the open probability, the open time, and the closed time of RyR models to higher Ca^2+^ concentrations. Decreased Mg^2+^ affinity had opposite effects. This is in line with the observed effect of Mg^2+^ on RyR calcium sensitivity (Laver et al., 1997; Gyorke and Gyorke, 1998; Zahradnikova et al., 2003). As originally revealed by Zahradnikova et al. (2010), the non-linear allosteric modulation of RyR activity by Mg^2+^ affected *P*_*O*_ and *t*_*C*_ significantly at sub-micromolar Ca^2+^ concentrations but did not influence RyR activity at the micromolar range. A change of *f*_*Mg*_, which may result from metabolic regulation of the ryanodine receptor, may thus modulate the frequency of spontaneous RyR openings in diastolic myocytes by an order of magnitude without a change in the characteristics of calcium release events.

The kinetic description confirmed previous results of the equilibrium model of the RyR inhibition site (Zahradnikova et al., 2010) that the RyR open probability and the spark amplitude are shaped by modulation of Mg^2+^ affinity of the RyR inhibition site. Additionally, the kinetic description revealed substantial effects of rate constants (*k*_*IonMg*_ and *k*_*IoffMg*_) of the RyR inhibition site on the RyR open and closed times and their relationships to the spark termination, CRE frequency, and relative occurrence of visible and invisible CREs (see below).

To sum up, the MWC-Ca/Mg RyR model predicts that at the physiological cytosolic concentration of 1 mM, Mg^2+^ ions influence the single-channel activity of RyRs considerably. Magnesium ions act through their allosteric competition with Ca^2+^ at the RyR activation site as well as through their binding at the RyR inhibition site. This means that modification of the Mg-binding properties of RyR channels by pharmacological, metabolic, or structural changes in RyR monomers may substantially affect the calcium release function of cardiac myocytes.

### Calcium Release Events Generated by the Calcium Release Site Model With Monod-Wyman-Changeux-Ca/Mg Ryanodine Receptor Models

To assess the impact of Mg-modulation of RyR activity on cardiac calcium release, we applied the MWC-Ca/Mg RyR model to the model of the calcium release site developed previously (Iaparov et al., 2021). Interestingly, the three types of calcium release events that were observed in simulations of CRS models based on the two-state RyR model (Iaparov et al., 2021) occurred also in CRS models based on the MWC-Ca/Mg RyR model; however, the occurrence of sparks was much less frequent in the MWC-Ca/Mg RyR model. The difference between a two-state model and the full model tested in this work stems in part from the calcium-independent open time of the two-state model (Iaparov et al., 2021) and the calcium-dependent open time of the full model (Figure 5). Additionally, the probability that many RyRs would become active in a spark is low since the rate of Mg^2+^ unbinding from the calcium activation site is much slower than the rate of Ca^2+^ binding, in line with experiments (Zahradnikova et al., 2003) and simulations (Zahradnikova et al., 2010).

Simulations of the reference CRS model predict that a large fraction of spontaneous local calcium release events (quarks and blips) result from the activation of a subset of RyR channels in a CRS (Figure 4). When the *i*_*Ca*_ amplitude was small (0.15 or 0.25 pA), CRS models produced mostly quarks or blips. Individual records of blips were formed by 2 to 6 RyRs open at their peaks (at around 3 ms), and terminated fast, within 5 to 6 ms, by simple stochastic attrition (Stern and Cheng, 2004). When the *i*_*Ca*_ was higher (0.4 or 0.6 pA), CRS models produced similar quarks and blips as at lower *i*_*Ca*_ amplitudes but also a significant fraction of large sparks. With the reference parameter set, these sparks were formed by 7 to 13 RyRs (out of 20 in the CRS) open at their peaks (30 to 40 ms) and terminated very slowly (within 60 to 70 ms) by the attrition mechanism. However, the average rate of spark termination was very sensitive to the Mg-binding rate constants of the RyR inhibitory site (see below).

The RyR single-channel calcium current of 0.6 pA produced a higher amplitude of sparks but also a stronger calcium fluorescence signal (Figure 4A) so that blips were well over the detection threshold of intracellular calcium measurements and sparks were high (Δ*F*/*F*_0_ > 3) and prolonged. If real, such large calcium release flux would cause a rapid dissipation of the calcium gradient across SR membrane and fast termination of calcium release by a mechanism dubbed “induction decay” (Cannell et al., 2013) or “pernicious attrition” (Gillespie and Fill, 2013).

### Effect of Mg^2+^ Binding Parameters on Calcium Release Events

Variation of the calcium sensitivity of RyRs by altering binding and unbinding of Mg^2+^ to/from the RyR activation site led to dramatic changes in the occurrence of spontaneous calcium release events and the relative proportions of quarks and sparks, while it had only a small effect on the relative occurrence of blips. In CRS simulations, the times to peak of both blips and sparks were very sensitive to the rates of Mg-binding and unbinding at the activation site. Changes that facilitated Ca^2+^ binding to the activation site, i.e., faster Mg-unbinding or slower Mg-binding, shortened the time-to-peak of blips and sparks, while the opposite changes prolonged it (Figure 6A). These findings indicate that Mg^2+^ ions modulate also the kinetics of the experimentally observable calcium sparks, although these changes may be blunted by the relatively slow kinetics of calcium indicators.

The allosteric coefficient *f*_*Mg*_ characterizes the change in the energy needed for the closed-open transition in the RyR homotetramer upon binding of Mg^2+^. In the CRS simulations, the change of *f*_*Mg*_ markedly affected the closed time, not the open time, at low Ca^2+^ concentration but the fraction of blips changed only slightly while the fraction of sparks was strongly reduced at high *f*_*Mg*_. As a result, the frequency of experimentally observable CREs (*f*_*B*_ and/or *f*_*S*_, Table 4) would increase with decreasing *f*_*Mg*_, and vice versa, while their time courses would be affected modestly (Figure 6B). The amplitude of the observed calcium fluorescence signals of CREs would be thus affected only slightly.

Variation of the Mg^2+^-binding at the RyR inhibition site affected calcium release events profoundly (Figures 6C,F). Faster Mg-unbinding or slower Mg-binding, both of which increased the maximum *P*_*O*_, increased the relative occurrence and the maximum *N*_*O*_ of sparks but also completely suppressed spontaneous spark termination. At the level of observed fluorescence signals, this would transpire as a large increase in the frequency and amplitude of the experimentally observable CREs, and thus as full reliance of spark termination on SR depletion. Slower Mg-unbinding or faster Mg-binding at the inhibition site, which both decreased the maximum *P*_*O*_, suppressed the sparks almost completely and decreased their amplitude and duration substantially. Additionally, the blips became substantially longer, without a change in their amplitude, when Mg-unbinding was slower. It should be pointed out here that if Mg^2+^ inhibition was absent, activation of more than one RyR in the CRS always led to the activation of all RyRs (Figure 7A, lower right panel).

### The Role of Mg^2+^ Binding in the Effective Coupling Strength Between Ryanodine Receptors

In a previous study (Iaparov et al., 2021) we characterized the interaction between ryanodine receptors through coupling strength - a weighted product of RyR vicinity and single-channel calcium current. The use of a more complex model of ryanodine receptor gating opened the question of whether the coupling strength is still applicable for the explanation of CRS activity. To this end, we introduced the effective coupling strength that accounted for variable RyR gating parameters (Eq. 10). This included weighing the parameters for Mg^2+^ binding to the activation site, the allosteric factor of Mg^2+^ binding to the activation site, or the parameters for Mg^2+^ binding to the inhibition site. Due to the multifactorial nature of the results (178 different parameter combinations), it was not possible to use fitting for the determination of the weight factors, since it was not *a priori* known whether they follow the same Hill equations, in contrast to our previous study (Iaparov et al., 2021). The weight factors were found by the original method of maximizing the mutual information between the effective coupling strength and CRE characteristics (see Methods). The weights enabled the comparison of CRS models differing in RyR Mg-binding parameter values.

As documented in Figure 7, the effective coupling strength consolidated the characteristics of simulated calcium release events into relationships of a dose-response type. The relative occurrence of quarks, blips, and sparks followed approximately the same relationship on the effective coupling strength, independently of RyR model parameters. However, the dependence of the average amplitude of all events differed markedly between models in which parameters of the activation site and the inhibition site were varied. This was mainly due to the steep dependence of RyR maximum open probability on *K*_*IMg*_, leading to a steep increase of spark amplitude when *K*_*IMg*_ was increased and inhibition of RyR activity by Mg^2+^ was reduced, independent of coupling strength.

### Limitations

The CRS model used in this work does not account for the reduction of calcium gradient and the effect of depletion of SR calcium on the single-channel calcium current. This simplification was necessary to assess the effect of Mg–RyR interactions on the formation of calcium release events without the interference of parallel processes. Consequently, the simulated CREs differ from the real ones, especially in the case of large and prolonged sparks.

The CRS model also takes advantage of a simplified description of buffered calcium diffusion based on the instantaneous formation of the steady-state calcium gradient upon RyR opening (Naraghi and Neher, 1997). This approximation also assumes that calcium buffers do not saturate and therefore the build-up of calcium concentration at a RyR can be considered as additive in the case of multiple sources. More realistically, the free Ca^2+^ concentration in the dyadic gap temporarily reached higher values, which might locally saturate the buffers according to the reaction-diffusion models of narrow dyadic gaps (Soeller et al., 2007; Valent et al., 2007). Since the activation of RyRs is much slower than the build-up of calcium gradient (Soeller et al., 2007; Valent et al., 2007), the use of the Naraghi-Neher simplification should not weaken the conclusions of this study while allowing much faster calculations than the reaction-diffusion model of the dyadic gap (Valent et al., 2007).

The rate constant *k*_*AonMg*_ was set to 0.01 × *k*_*AonCa*_, considering that the rate-limiting factor in ion binding to a ligand is the exchange rate of a water molecule in the hydration complex of the ion. The resulting value can be considered as the upper limit since other rate-limiting factors in Mg-RyR interaction could eventually slow down the reaction. Consequently, the corresponding unbinding rate constant *k*_*AoffMg*_ might have been overestimated by the fitting procedure to keep the equilibrium constant at the appropriate value.

We have previously estimated *k*_*AoffMg*_ in the range of 0.05 - 0.22 ms^–1^, assuming instantaneous binding of Ca^2+^ to an Mg^2+^-free RyR activation site (Zahradnikova et al., 2010). The known rate constants of Mg^2+^ unbinding from other calcium-binding proteins such as parvalbumin, its mutants, and troponin-C range widely between 0.001 ms^–1^ to 0.267 ms^–1^ (Rosenfeld and Taylor, 1985; Permyakov et al., 1987; Hou et al., 1993; Zhang et al., 2011), but are less than our estimate. We showed by permutation of Mg^2+^ binding/unbinding rates at a constant ratio that the lower rates (at no change in Mg^2+^ affinity) would have no effects on the calcium dependence of the steady-state *P*_*O*_, *t*_*O*_ and *t*_*C*_ (Figure 5) and would only affect the time course of the response to a stepwise change in [Ca^2+^]. This simultaneous and equivalent decrease of both rate constants would result in a still lower frequency of observable events (Figure 7B and Table 4).

In general, the rates of ligand-ion binding reactions are temperature dependent; however, the temperature coefficients of similar reaction systems are comparable but larger than that of passive diffusion, and smaller than that of enzymatic reactions. Therefore, their proportions should be expected to change with the temperature of the heart. Nevertheless, experiments at the cellular level are typically performed at room temperature, for which the presented results are pertinent.

Finally, our results point to the need for a more precise single-channel description of RyR activity at near-physiological conditions, especially at low cytosolic [Ca^2+^], in the presence of high ATP, and at a range of free Mg^2+^ concentrations. Such measurements would need very long recordings of single-channel activity to attain sufficient accuracy (Zahradnikova et al., 2020) but would be instrumental for a precise determination of *K_*O*0_* and *f*_*Mg*_.

### Physiological Implications

The presented model provided a high fraction of quarks, i.e., initiating RyR openings that activated no other RyRs. In real experiments, quarks are not observable for their small calcium yield. To determine whether this finding is consistent with experimental data, we compared the predicted fraction of CREs of different types with the observed number of calcium release events in cardiac myocytes. The number of ryanodine receptors per volume unit of a myocyte has been estimated as 60 - 90 RyRs per μm^3^ (Soeller et al., 2007; Hayashi et al., 2009). Assuming a confocal volume of 100 × 1 × 0.2 = 20 μm^3^ per scanning line, this translates to 1250 - 1900 RyRs in the observation volume per 100 μm line length. Our reference value of mean closed time (35 s) and mean open time (0.2 ms) at 100 nM Ca^2+^ then corresponds to 36 - 54 RyR openings per second in this volume. Of these, there would be 2.7 - 8.4 blips per second and 0.02 - 7 sparks per second at a calcium current of 0.15 - 0.6 pA (see Figure 4D and Table 4). These can be compared with the experimental values of 0.2 - 4.6 sparks/(100 μm) (Lukyanenko and Gyorke, 1999; Guo et al., 2006; Parks and Howlett, 2012; Yin et al., 2021) in intact cells and 4 - 40 sparks/(100 μm) in skinned cardiac myocytes (Lukyanenko and Gyorke, 1999; Kubalova et al., 2005; Fernandez-Velasco et al., 2009; Ruiz-Hurtado et al., 2015). The values predicted by the model can be reconciled either with a lower *i*_*Ca*_ and a high predominance of blips or with a higher *i*_*Ca*_ and a comparable occurrence of both blips and sparks. However, our simplified calculations predict that blips cannot be detected when *i*_*Ca*_ is lower than 0.4 pA (Iaparov et al., 2021); thus a more realistic model of calcium diffusion in the dyadic gap and better estimates of Mg^2+^-ATP binding kinetics are necessary to decide this point.

The Mg-binding parameters substantially affect the frequency of RyR openings by modulating the RyR closed time at the basal calcium concentration (Table 4). A similar conclusion was reached in our previous study of the effect of Mg^2+^ binding on spontaneous sparks and waves in a simplified kinetic model of the CRS (Petrovic et al., 2015). Additionally, here we observed a strong effect of Mg-binding parameters on the relative occurrence of quarks, blips, and sparks (Figures 6D-F). Such changes transposed to real experiments mean that a change in Mg-binding parameters due to RyR mutation, phosphorylation/dephosphorylation, or oxidation/reduction would affect the frequency of observed spontaneous calcium release events. An increase in the relative proportion of sparks would be associated with an increase of the mean amplitude of the observed CREs, due to the larger number of open RyRs at the peak of sparks than of blips, and a longer average duration of observed CREs, due to the longer duration of sparks than blips (Figure 4D).

## Conclusion

The binding of Mg^2+^ to both the activation and the inhibition site negatively modulated the effective coupling strength in a qualitatively similar way. In other words, these findings reveal the role of Mg^2+^ ions as a damping agent that protects calcium release sites from uncontrolled activation at threshold calcium stimuli. This finding is of principal physiological importance since it helps to explain the graded behavior of calcium release at the level of ryanodine receptors, which impedes the positive feedback inherent to the calcium-induced calcium release mechanism.

## Data Availability Statement

The raw data supporting the conclusions of this article will be made available by the authors, without undue reservation.

## Author Contributions

BI, IZ, and AZ planned the research and wrote the manuscript. BI and AZ designed the simulations. BI wrote the software, derived mathematical expressions, and performed simulations. BI, JB, IZ, and AZ analyzed and interpreted the experimental and simulated data. All authors contributed to the article and approved the submitted version.

## Conflict of Interest

The authors declare that the research was conducted in the absence of any commercial or financial relationships that could be construed as a potential conflict of interest.

## Publisher’s Note

All claims expressed in this article are solely those of the authors and do not necessarily represent those of their affiliated organizations, or those of the publisher, the editors and the reviewers. Any product that may be evaluated in this article, or claim that may be made by its manufacturer, is not guaranteed or endorsed by the publisher.

## Footnotes

1
https://www.openmp.org

## Supplementary Material

The derivation of the equations used in Methods is given in the Supplementary Material. The transition rates used for constructing the Q-matrix are summarized in Supplementary Table 1. The values of Mg-binding parameters used in this study are given in Supplementary Table 2.

## References

[B1] Bognar M. (2013). *OMPRNG: A Parallel Statistical Random Number Generator for OpenMP.* Available online at: https://web.archive.org/web/20180125041447/http://homepage.divms.uiowa.edu/~mbognar/omprng/ (accessed December 29, 2021).

[B2] Bovo E. Mazurek S. R. Blatter L. A. Zima A. V. (2011). Regulation of sarcoplasmic reticulum Ca(2)(+) leak by cytosolic Ca(2)(+) in rabbit ventricular myocytes. *J. Physiol.* 589(Pt 24), 6039–6050. 10.1113/jphysiol.2011.214171 21986204 PMC3286684

[B3] Bridge J. H. Ershler P. R. Cannell M. B. (1999). Properties of Ca2+ sparks evoked by action potentials in mouse ventricular myocytes. *J. Physiol.* 518 469–478. 10.1111/j.1469-7793.1999.0469p.x 10381593 PMC2269442

[B4] Cannell M. B. Kong C. H. Imtiaz M. S. Laver D. R. (2013). Control of sarcoplasmic reticulum Ca2+ release by stochastic RyR gating within a 3D model of the cardiac dyad and importance of ‘induction decay’ for CICR termination. *Biophys. J.* 104 2149–2159. 10.1016/j.bpj.2013.03.058 23708355 PMC3660628

[B5] Cheng H. Lederer W. J. (2008). Calcium sparks. *Physiol. Rev.* 88 1491–1545. 18923188 10.1152/physrev.00030.2007

[B6] Chi X. Gong D. Ren K. Zhou G. Huang G. Lei J. (2019). Molecular basis for allosteric regulation of the type 2 ryanodine receptor channel gating by key modulators. *Proc. Natl. Acad. Sci. U.S.A.* 116 25575–25582. 10.1073/pnas.1914451116 31792195 PMC6926060

[B7] Choi R. H. Koenig X. Launikonis B. S. (2017). Dantrolene requires Mg(2+) to arrest malignant hyperthermia. *Proc. Natl. Acad. Sci. U.S.A.* 114 4811–4815. 10.1073/pnas.1619835114 28373535 PMC5422773

[B8] Colquhoun D. Hawkes A. G. (1995). “A Q-matrix cookbook. How to write only one program to calculate the single-channel and macroscopic predictions for any kinetic mechanism,” in *Single-Channel Recording*, second Edn, eds Sakmann B. Neher E. (New York, NY: Plenum Press), 589–633. 10.1007/978-1-4419-1229-9_20

[B9] Copello J. A. Barg S. Sonnleitner A. Porta M. Diaz-Sylvester P. Fill M. (2002). Differential activation by Ca2+, ATP and caffeine of cardiac and skeletal muscle ryanodine receptors after block by Mg2+. *J. Membr. Biol.* 187 51–64. 10.1007/s00232-001-0150-x 12029377

[B10] Dashti A. Mashayekhi G. Shekhar M. Ben Hail D. Salah S. Schwander P. (2020). Retrieving functional pathways of biomolecules from single-particle snapshots. *Nat. Commun.* 11:4734. 10.1038/s41467-020-18403-x 32948759 PMC7501871

[B11] des Georges A. Clarke O. B. Zalk R. Yuan Q. Condon K. J. Grassucci R. A. (2016). Structural basis for gating and activation of RyR1. *Cell* 167 145–157. 10.1016/j.cell.2016.08.075 27662087 PMC5142848

[B12] Eigen M. Wilkins R. G. (eds) (1965). “The kinetics and mechanism of formation of metal complexes,” in *Mechanisms of Inorganic Reactions.* (Washington, D.C: American Chemical Society), 55–80.

[B13] Fabiato A. (1983). Calcium-induced release of calcium from the cardiac sarcoplasmic reticulum. *Am. J. Physiol.* 245 C1–C14. 6346892 10.1152/ajpcell.1983.245.1.C1

[B14] Fernandez-Velasco M. Rueda A. Rizzi N. Benitah J. P. Colombi B. Napolitano C. (2009). Increased Ca2+ sensitivity of the ryanodine receptor mutant RyR2R4496C underlies catecholaminergic polymorphic ventricular tachycardia. *Circ. Res.* 104 201–209. 10.1161/CIRCRESAHA.108.177493 19096022 PMC2796688

[B15] Gillespie D. Fill M. (2013). Pernicious attrition and inter-RyR2 CICR current control in cardiac muscle. *J. Mol. Cell Cardiol.* 58 53–58. 10.1016/j.yjmcc.2013.01.011 23369697 PMC3628281

[B16] Gillespie D. T. (1977). Exact stochastic simulation of coupled chemical reactions. *J. Phys. Chem.* 81 2340–2391. 10.1021/j100540a008

[B17] Guo T. Zhang T. Mestril R. Bers D. M. (2006). Ca2+/Calmodulin-dependent protein kinase II phosphorylation of ryanodine receptor does affect calcium sparks in mouse ventricular myocytes. *Circ. Res.* 99 398–406. 10.1161/01.RES.0000236756.06252.13 16840718

[B18] Guo W. Sun B. Estillore J. P. Wang R. Chen S. R. W. (2020). The central domain of cardiac ryanodine receptor governs channel activation, regulation, and stability. *J. Biol. Chem.* 295 15622–15635. 10.1074/jbc.RA120.013512 32878990 PMC7667974

[B19] Gusev K. Niggli E. (2008). Modulation of the local SR Ca2+ release by intracellular Mg2+ in cardiac myocytes. *J. Gen. Physiol.* 132 721–730. 10.1085/jgp.200810119 19029377 PMC2585859

[B20] Gyorke I. Gyorke S. (1998). Regulation of the cardiac ryanodine receptor channel by luminal Ca2+ involves luminal Ca2+ sensing sites. *Biophys. J.* 75 2801–2810. 10.1016/S0006-3495(98)77723-9 9826602 PMC1299953

[B21] Hake J. Edwards A. G. Yu Z. Kekenes-Huskey P. M. Michailova A. P. McCammon J. A. (2012). Modelling cardiac calcium sparks in a three-dimensional reconstruction of a calcium release unit. *J. Physiol.* 590 4403–4422. 10.1113/jphysiol.2012.227926 22495592 PMC3477749

[B22] Harris C. R. Millman K. J. van der Walt S. J. Gommers R. Virtanen P. Cournapeau D. (2020). Array programming with NumPy. *Nature* 585 357–362. 10.1038/s41586-020-2649-2 32939066 PMC7759461

[B23] Hartmann N. Pabel S. Herting J. Schatter F. Renner A. Gummert J. (2017). Antiarrhythmic effects of dantrolene in human diseased cardiomyocytes. *Heart Rhythm.* 14 412–419. 10.1016/j.hrthm.2016.09.014 27650424

[B24] Hayashi T. Martone M. E. Yu Z. Thor A. Doi M. Holst M. J. (2009). Three-dimensional electron microscopy reveals new details of membrane systems for Ca2+ signaling in the heart. *J. Cell Sci.* 122(Pt 7), 1005–1013. 10.1242/jcs.028175 19295127 PMC2720931

[B25] Hou T. T. Johnson J. D. Rall J. A. (1993). Role of parvalbumin in relaxation of frog skeletal muscle. *Adv. Exp. Med. Biol.* 332 141–151. 10.1007/978-1-4615-2872-2_13 8109327

[B26] Iaparov B. I. Zahradnik I. Moskvin A. S. Zahradnikova A. (2021). In silico simulations reveal that RYR distribution affects the dynamics of calcium release in cardiac myocytes. *J. Gen. Physiol.* 153:e202012685. 10.1085/jgp.202012685 33735373 PMC7980188

[B27] Janicek R. Zahradnikova A. Jr. Polakova E. Pavelkova J. Zahradnik I. (2012). Calcium spike variability in cardiac myocytes results from activation of small cohorts of ryanodine receptor 2 channels. *J. Physiol.* 590 5091–5106. 10.1113/jphysiol.2012.234823 22890710 PMC3497565

[B28] Jayasinghe I. Clowsley A. H. Lin R. Lutz T. Harrison C. Green E. (2018). True molecular scale visualization of variable clustering properties of ryanodine receptors. *Cell Rep.* 22 557–567. 10.1016/j.celrep.2017.12.045 29320748 PMC5775502

[B29] Kolstad T. R. van den Brink J. MacQuaide N. Lunde P. K. Frisk M. Aronsen J. M. (2018). Ryanodine receptor dispersion disrupts Ca(2+) release in failing cardiac myocytes. *eLife* 7:e39427. 10.7554/eLife.39427 30375974 PMC6245731

[B30] Kraskov A. Stogbauer H. Grassberger P. (2004). Estimating mutual information. *Phys. Rev. E* 69:066138. 10.1103/Physreve.69.066138 15244698

[B31] Kubalova Z. Terentyev D. Viatchenko-Karpinski S. Nishijima Y. Gyorke I. Terentyeva R. (2005). Abnormal intrastore calcium signaling in chronic heart failure. *Proc. Natl. Acad. Sci. U.S.A.* 102 14104–14109. 10.1073/pnas.0504298102 16172392 PMC1236548

[B32] Kunze D. L. Lacerda A. E. Wilson D. L. (1985). Cardiac Na currents and the inactivating, reopening, and waiting properties of single cardiac Na channels. *J. Gen. Physiol.* 86 691–719. 10.1085/jgp.86.5.691 2415670 PMC2228812

[B33] Laver D. R. Baynes T. M. Dulhunty A. F. (1997). Magnesium inhibition of ryanodine-receptor calcium channels: evidence for two independent mechanisms. *J. Membr. Biol.* 156 213–229. 10.1007/s002329900202 9096063

[B34] Lehnart S. E. Wehrens X. H. Laitinen P. J. Reiken S. R. Deng S. X. Cheng Z. (2004). Sudden death in familial polymorphic ventricular tachycardia associated with calcium release channel (ryanodine receptor) leak. *Circulation* 109 3208–3214. 10.1161/01.CIR.0000132472.98675.EC 15197150

[B35] Li J. Imtiaz M. S. Beard N. A. Dulhunty A. F. Thorne R. vanHelden D. F. (2013). ss-Adrenergic stimulation increases RyR2 activity via intracellular Ca2+ and Mg2+ regulation. *PLoS One* 8:e58334. 10.1371/journal.pone.0058334 23533585 PMC3606165

[B36] Li P. Chen S. R. (2001). Molecular basis of Ca(2)+ activation of the mouse cardiac Ca(2)+ release channel (ryanodine receptor). *J. Gen. Physiol.* 118 33–44. 10.1085/jgp.118.1.33 11429443 PMC2233748

[B37] Lukyanenko V. Gyorke I. Subramanian S. Smirnov A. Wiesner T. F. Gyorke S. (2000). Inhibition of Ca(2+) sparks by ruthenium red in permeabilized rat ventricular myocytes. *Biophys. J.* 79 1273–1284. 10.1016/S0006-3495(00)76381-8 10968991 PMC1301023

[B38] Lukyanenko V. Gyorke S. (1999). Ca2+ sparks and Ca2+ waves in saponin-permeabilized rat ventricular myocytes. *J. Physiol.* 521(Pt 3), 575–585. 10.1111/j.1469-7793.1999.00575.x 10601490 PMC2269691

[B39] Lukyanenko V. Viatchenko-Karpinski S. Smirnov A. Wiesner T. F. Gyorke S. (2001). Dynamic regulation of sarcoplasmic reticulum Ca(2+) content and release by luminal Ca(2+)-sensitive leak in rat ventricular myocytes. *Biophys. J.* 81 785–798. 10.1016/S0006-3495(01)75741-4 11463625 PMC1301553

[B40] Macquaide N. Tuan H. T. Hotta J. Sempels W. Lenaerts I. Holemans P. (2015). Ryanodine receptor cluster fragmentation and redistribution in persistent atrial fibrillation enhance calcium release. *Cardiovasc. Res.* 108 387–398. 10.1093/cvr/cvv231 26490742 PMC4648199

[B41] MacQueen J. (1967). “Some methods for classification and analysis of multivariate observations,” in *Proceedings of the Fifth Berkeley Symposium on Mathematical Statistics and Probability*, eds Le Cam L. M. Neyman J. (Berkeley: University of California Press).

[B42] Marx S. O. Reiken S. Hisamatsu Y. Jayaraman T. Burkhoff D. Rosemblit N. (2000). PKA phosphorylation dissociates FKBP12.6 from the calcium release channel (ryanodine receptor): defective regulation in failing hearts. *Cell* 101 365–376. 10.1016/s0092-8674(00)80847-8 10830164

[B43] Matveev V. Sherman A. Zucker R. S. (2002). New and corrected simulations of synaptic facilitation. *Biophys. J.* 83 1368–1373. 10.1016/S0006-3495(02)73907-6 12202362 PMC1302235

[B44] McKinney W. (2010). “Data structures for statistical computing in python,” in *Proceedings of the 9th Python in Science Conference*, Austin, TX.

[B45] Meissner G. (1994). Ryanodine receptor/Ca2+ release channels and their regulation by endogenous effectors. *Annu. Rev. Physiol.* 56 485–508. 10.1146/annurev.ph.56.030194.002413 7516645

[B46] Meissner G. (2004). Molecular regulation of cardiac ryanodine receptor ion channel. *Cell Calcium* 35 621–628. 10.1016/j.ceca.2004.01.015 15110152

[B47] Munro M. L. van Hout I. Aitken-Buck H. M. Sugunesegran R. Bhagwat K. Davis P. J. (2021). Human atrial fibrillation is not associated with remodeling of ryanodine receptor clusters. *Front. Cell Dev. Biol.* 9:633704. 10.3389/fcell.2021.633704 33718369 PMC7947344

[B48] Naraghi M. Neher E. (1997). Linearized buffered Ca2+ diffusion in microdomains and its implications for calculation of [Ca2+] at the mouth of a calcium channel. *J. Neurosci.* 17 6961–6973. 10.1523/JNEUROSCI.17-18-06961.1997 9278532 PMC6573285

[B49] Newville M. Stensitzki T. Allen D. B. Ingargiola A. (2014). *LMFIT: Non-Linear Least-Square Minimization and Curve-Fitting for Python.* San Francisco, CA: GitHub.

[B50] Niggli E. Shirokova N. (2007). A guide to sparkology: the taxonomy of elementary cellular Ca2+ signaling events. *Cell Calcium* 42 379–387. 10.1016/j.ceca.2007.02.010 17428535

[B51] Okuda S. Sufu-Shimizu Y. Kato T. Fukuda M. Nishimura S. Oda T. (2018). CaMKII-mediated phosphorylation of RyR2 plays a crucial role in aberrant Ca(2+) release as an arrhythmogenic substrate in cardiac troponin T-related familial hypertrophic cardiomyopathy. *Biochem. Biophys. Res. Commun.* 496 1250–1256. 10.1016/j.bbrc.2018.01.181 29402414

[B52] Parks R. J. Howlett S. E. (2012). H-89 decreases the gain of excitation-contraction coupling and attenuates calcium sparks in the absence of beta-adrenergic stimulation. *Eur. J. Pharmacol.* 691 163–172. 10.1016/j.ejphar.2012.07.012 22796673

[B53] Pedregosa F. Varoquaux G. Gramfort A. Michel V. Thirion B. Grisel O. (2011). Scikit-learn: machine learning in python. *J. Mach. Learn. Res.* 12 2825–2830. 10.1080/13696998.2019.1666854 31505982

[B54] Permyakov E. A. Ostrovsky A. V. Kalinichenko L. P. (1987). Stopped-flow kinetic studies of Ca(II) and Mg(II) dissociation in cod parvalbumin and bovine alpha-lactalbumin. *Biophys. Chem.* 28 225–233. 10.1016/0301-4622(87)80093-5 3440123

[B55] Petrovic P. Valent I. Cocherova E. Pavelkova J. Zahradnikova A. (2015). Ryanodine receptor gating controls generation of diastolic calcium waves in cardiac myocytes. *J. Gen. Physiol.* 145 489–511. 10.1085/jgp.201411281 26009544 PMC4442793

[B56] Rosenfeld S. S. Taylor E. W. (1985). Kinetic studies of calcium and magnesium binding to troponin C. *J. Biol. Chem.* 260 242–251. 3965449

[B57] Rousseau E. Smith J. S. Henderson J. S. Meissner G. (1986). Single channel and 45 Ca2+ flux measurements of the cardiac sarcoplasmic reticulum calcium channel. *Biophys. J.* 50 1009–1014. 10.1016/s0006-3495(86)83543-3 2431724 PMC1329827

[B58] Ruiz-Hurtado G. Li L. Fernandez-Velasco M. Rueda A. Lefebvre F. Wang Y. (2015). Reconciling depressed Ca2+ sparks occurrence with enhanced RyR2 activity in failing mice cardiomyocytes. *J. Gen. Physiol.* 146 295–306. 10.1085/jgp.201511366 26371209 PMC4586588

[B59] Shang W. Lu F. Sun T. Xu J. Li L. L. Wang Y. (2014). Imaging Ca2+ nanosparks in heart with a new targeted biosensor. *Circ. Res.* 114 412–420. 10.1161/CIRCRESAHA.114.302938 24257462

[B60] Shannon C. E. (1948). A mathematical theory of communication. *Bell Syst. Tech. J.* 27 379–423. 10.1002/j.1538-7305.1948.tb01338.x

[B61] Smith G. D. Keizer J. E. Stern M. D. Lederer W. J. Cheng H. (1998). A simple numerical model of calcium spark formation and detection in cardiac myocytes. *Biophys. J.* 75 15–32. 10.1016/S0006-3495(98)77491-0 9649364 PMC1299676

[B62] Soeller C. Crossman D. Gilbert R. Cannell M. B. (2007). Analysis of ryanodine receptor clusters in rat and human cardiac myocytes. *Proc. Natl. Acad. Sci. U.S.A.* 104 14958–14963. 10.1073/pnas.0703016104 17848521 PMC1986595

[B63] Steele D. S. Duke A. M. (2007). Defective Mg2+ regulation of RyR1 as a causal factor in malignant hyperthermia. *Arch. Biochem. Biophys.* 458 57–64. 10.1016/j.abb.2006.03.001 16620769

[B64] Stern M. D. (1992). Theory of excitation - contraction coupling in cardiac muscle. *Biophys. J.* 63 497–517. 10.1016/s0006-3495(92)81615-6 1330031 PMC1262173

[B65] Stern M. D. Cheng H. (2004). Putting out the fire: what terminates calcium-induced calcium release in cardiac muscle? *Cell Calcium* 35 591–601. 10.1016/j.ceca.2004.01.013 15110149

[B66] Storn R. Price K. (1997). Differential evolution - A simple and efficient heuristic for global optimization over continuous spaces. *J. Glob. Optim.* 11 341–359. 10.1023/A:1008202821328

[B67] Tencerova B. Zahradnikova A. Gaburjakova J. Gaburjakova M. (2012). Luminal Ca2+ controls activation of the cardiac ryanodine receptor by ATP. *J. Gen. Physiol.* 140 93–108. 10.1085/jgp.201110708 22851674 PMC3409101

[B68] Valent I. Zahradnikova A. Pavelkova J. Zahradnik I. (2007). Spatial and temporal Ca2+, Mg2+, and ATP2- dynamics in cardiac dyads during calcium release. *Biochim. Biophys. Acta* 1768 155–166. 10.1016/j.bbamem.2006.08.020 17034755

[B69] Virtanen P. Gommers R. Oliphant T. E. Haberland M. Reddy T. Cournapeau D. (2020). SciPy 1.0: fundamental algorithms for scientific computing in Python. *Nat. Methods* 17 261–272. 10.1038/s41592-019-0686-2 32015543 PMC7056644

[B70] Walker M. A. Kohl T. Lehnart S. E. Greenstein J. L. Lederer W. J. Winslow R. L. (2015). On the adjacency matrix of RyR2 cluster structures. *PLoS Comput. Biol.* 11:e1004521. 10.1371/journal.pcbi.1004521 26545234 PMC4636394

[B71] Wang S. Q. Stern M. D. Rios E. Cheng H. (2004). The quantal nature of Ca2+ sparks and in situ operation of the ryanodine receptor array in cardiac cells. *Proc. Natl. Acad. Sci. U.S.A.* 101 3979–3984. 10.1073/pnas.0306157101 15004280 PMC374355

[B72] Xu L. Mann G. Meissner G. (1996). Regulation of cardiac Ca2+ release channel (ryanodine receptor) by Ca2+, H+, Mg2+, and adenine nucleotides under normal and simulated ischemic conditions. *Circ. Res.* 79 1100–1109. 10.1161/01.res.79.6.1100 8943948

[B73] Yin L. Zahradnikova A. Jr. Rizzetto R. Boncompagni S. Rabesahala de Meritens C. (2021). Impaired binding to Junctophilin-2 and nanostructural alteration in CPVT mutation. *Circ. Res.* 129 e35–e52. 10.1161/CIRCRESAHA.121.319094 34111951 PMC8320243

[B74] Zahradnik I. Gyorke S. Zahradnikova A. (2005). Calcium activation of ryanodine receptor channels–reconciling RyR gating models with tetrameric channel structure. *J. Gen. Physiol.* 126 515–527. 10.1085/jgp.200509328 16260840 PMC2266604

[B75] Zahradnikova A. Jr. Polakova E. Zahradnik I. Zahradnikova A. (2007). Kinetics of calcium spikes in rat cardiac myocytes. *J. Physiol.* 578(Pt 3), 677–691. 10.1113/jphysiol.2006.117796 17124272 PMC2151335

[B76] Zahradnikova A. Dura M. Gyorke I. Escobar A. L. Zahradnik I. Gyorke S. (2003). Regulation of dynamic behavior of cardiac ryanodine receptor by Mg2+ under simulated physiological conditions. *Am. J. Physiol.* 285 C1059–C1070. 10.1152/ajpcell.00118.2003 12839831

[B77] Zahradnikova A. Iaparov B. Zahradnik I. (2020). The problem of accuracy in single-channel open probability measurements. *Prog. Biophys. Mol. Biol.* 157 94–106. 10.1016/j.pbiomolbio.2020.05.002 32416189

[B78] Zahradnikova A. Valent I. Zahradnik I. (2010). Frequency and release flux of calcium sparks in rat cardiac myocytes: a relation to RYR gating. *J. Gen. Physiol.* 136 101–116. 10.1085/jgp.200910380 20548054 PMC2894546

[B79] Zahradnikova A. Zahradnik I. (2012). Construction of calcium release sites in cardiac myocytes. *Front. Physiol.* 3:322. 10.3389/fphys.2012.00322 22934071 PMC3429091

[B80] Zahradnikova A. Zahradnik I. Gyorke I. Gyorke S. (1999). Rapid activation of the cardiac ryanodine receptor by submillisecond calcium stimuli. *J. Gen. Physiol.* 114 787–798. 10.1085/jgp.114.6.787 10578015 PMC2230654

[B81] Zhang J. Shettigar V. Zhang G. C. Kindell D. G. Liu X. Lopez J. J. (2011). Engineering Parvalbumin for the Heart: optimizing the Mg binding properties of rat beta-Parvalbumin. *Front. Physiol.* 2:77. 10.3389/fphys.2011.00077 22059076 PMC3204457

