# Supplementary material for: Magnesium Ions Moderate Calcium-Induced Calcium Release in Cardiac Calcium Release Sites by Binding to Ryanodine Receptor Activation and Inhibition Sites
Source: Front Physiol. 2022 Jan 25;12:805956. doi: 10.3389/fphys.2021.805956 (PMC8821920; doi:10.3389/fphys.2021.805956)
Supplement: Supplementary file 1 [file Table_1.pdf]

# Magnesium ions moderate calcium-induced calcium release in cardiac calcium release sites by binding to ryanodine receptor activation and inhibition sites

**Bogdan Iaparov, Iuliia Baglaeva, Ivan Zahradník, Alexandra Zahradníková**

Institute of Experimental Endocrinology, Department of Cellular Cardiology, Biomedical Research Center of the Slovak Academy of Sciences, Bratislava, Slovak Republic

## *Supplementary Material*

## 1 Supplementary Data

### 1.1 Derivation of the equation for RyR open probability calculations

The RyR channel was described as an MWC-type homotetrameric molecule with one activation site on each monomer, for which $Ca^{2+}$ and $Mg^{2+}$ ions compete, and with one common inhibition site that binds two $Mg^{2+}$ ions (Figure 2 of the main text).

Equation 11 from the main text was derived using the statistical mechanics framework (Marzen et al., 2013). Each state of the RyR molecule (Figure 2 of the main text) can be denoted as $X_{i,j,k}$, where $X \in \{C, O\}$ is the macrostate of the channel (open or closed), *i* and *j* are the numbers of bound $Ca^{2+}$ and $Mg^{2+}$ ions to the RyR activation site, respectively, and *k* is the number of magnesium $Mg^{2+}$ ions bound to the RyR inhibition site. The energy of a molecule can be decomposed into the conformational energy and the binding energies of ligands. In a homotetrameric molecule of the MWC type, the conformational energy of each monomer is the same and corresponds to the state of the molecule as a whole (in our case, open or closed). The binding energies depend only on the macrostate *X*. We considered the same binding energy for the RyR activation site of all monomers, which leads to the existence of several equivalent microstates, i.e., there are several combinations of the number of *i* calcium and *j* magnesium ions bound to the four equal activation binding sites. Disregarding the RyR interaction with $Mg^{2+}$ at the RyR inhibition site, which will be added to the final equation later, we can write the energy of the molecule in the state $X_{i,j}$ as follows:

$$E_{X_{i,j}} = E_X + iE_{b_X}^{Ca^{2+}} + jE_{b_X}^{Mg^{2+}}, \tag{S1}$$

where $E_X$ is the conformational energy in the macrostate $X_{i,j}$, $E_{b_X}^{Ca^{2+}}$ and $E_{b_X}^{Mg^{2+}}$ are the binding energies of calcium and magnesium ions when the molecule is in the macrostate *X*. The weight $w_{X_{i,j}}$ of each state equals to $g(X_{i,j})e^{-\beta(E_{X_{i,j}}-(i\mu_{Ca^{2+}}+j\mu_{Mg^{2+}}))}$ where $\beta = \frac{1}{k_B T}$, $\mu$ is the chemical potential (the energy cost of moving a ligand molecule from the solution to the receptor) and $g(X_{i,j})$ is the number of microstates of the state $X_{i,j}$, which in our case is the number of ways to put *i* calcium and *j* magnesium ions on the binding sites of the four monomers. The value of $g(X_{i,j})$ is calculated as the product of the respective binomial coefficients:

$$g(X_{i,j}) = \binom{4}{k}\binom{4-k}{l}. \tag{S2}$$

The chemical potential $\mu$ depends on the ligand concentration as follows:

$$\mu_c = \mu_{c_{ref}} + \frac{1}{\beta}\ln\frac{c}{c_{ref}}, \tag{S3}$$

where $c$ is the concentration, $c_{ref}$ is the reference concentration, and $\mu_{c_{ref}}$ is the reference chemical potential of the ligand. Combining equations S1-S3, the weight equals to:

$$w_{X_{i,j}} = \binom{4}{i}\binom{4-i}{j}\left(\frac{[Ca^{2+}]}{[Ca^{2+}]_{ref}}\right)^i\left(\frac{[Mg^{2+}]}{[Mg^{2+}]_{ref}}\right)^j e^{-\beta(E_X+i(E_{b_X}^{Ca^{2+}}-\mu_{Ca^{2+}_{ref}})+j(E_{b_X}^{Mg^{2+}}-\mu_{Mg^{2+}_{ref}}))} \tag{S4}$$

Open probability is the fraction of weights of open states:

$$P_O = \frac{w_{open}}{w_{open}+w_{closed}}. \tag{S5}$$

The weight of a macrostate is the sum of weights of all corresponding microstates. These weights can be calculated as follows:

$$w_{open} = e^{-\beta E_O}\sum_{i=0}^{4}\sum_{j=0}^{4-i}\binom{4}{i}\binom{4-i}{j}\left(\frac{[Ca^{2+}]}{[Ca^{2+}]_{ref}}\right)^i\left(\frac{[Mg^{2+}]}{[Mg^{2+}]_{ref}}\right)^j e^{-\beta(i(E_{b_O}^{Ca^{2+}}-\mu_{Ca^{2+}_{ref}})+j(E_{b_O}^{Mg^{2+}}-\mu_{Mg^{2+}_{ref}}))}$$

$$= e^{-\beta E_O}\left(1+\frac{[Ca^{2+}]}{[Ca^{2+}]_{ref}}e^{-\beta\left(E_{b_O}^{Ca^{2+}}-\mu_{Ca^{2+}_{ref}}\right)}+\frac{[Mg^{2+}]}{[Mg^{2+}]_{ref}}e^{-\beta\left(E_{b_O}^{Mg^{2+}}-\mu_{Mg^{2+}_{ref}}\right)}\right)^4, \tag{S6}$$

$$w_{closed} = e^{-\beta E_C}\sum_{i=0}^{4}\sum_{j=0}^{4-i}\binom{4}{i}\binom{4-i}{j}\left(\frac{[Ca^{2+}]}{[Ca^{2+}]_{ref}}\right)^i\left(\frac{[Mg^{2+}]}{[Mg^{2+}]_{ref}}\right)^j e^{-\beta(i(E_{b_C}^{Ca^{2+}}-\mu_{Ca^{2+}_{ref}})+j(E_{b_C}^{Mg^{2+}}-\mu_{Mg^{2+}_{ref}}))}$$

$$= e^{-\beta E_C}\left(1+\frac{[Ca^{2+}]}{[Ca^{2+}]_{ref}}e^{-\beta\left(E_{b_C}^{Ca^{2+}}-\mu_{Ca^{2+}_{ref}}\right)}+\frac{[Mg^{2+}]}{[Mg^{2+}]_{ref}}e^{-\beta\left(E_{b_C}^{Mg^{2+}}-\mu_{Mg^{2+}_{ref}}\right)}\right)^4. \tag{S7}$$

From these equations, the channel open probability $P_O$ at different concentrations of $Ca^{2+}$ and $Mg^{2+}$ in the absence of the RyR inhibition site can be calculated as:

$$P_O([Ca^{2+}],[Mg^{2+}]) = \frac{e^{-\beta E_O}\left(1+\frac{[Ca^{2+}]}{[Ca^{2+}]_{ref}}e^{-\beta\left(E_{b_O}^{Ca^{2+}}-\mu_{Ca^{2+}_{ref}}\right)}+\frac{[Mg^{2+}]}{[Mg^{2+}]_{ref}}e^{-\beta\left(E_{b_O}^{Mg^{2+}}-\mu_{Mg^{2+}_{ref}}\right)}\right)^4}{e^{-\beta E_O}\left(1+\frac{[Ca^{2+}]}{[Ca^{2+}]_{ref}}e^{-\beta\left(E_{b_O}^{Ca^{2+}}-\mu_{Ca^{2+}_{ref}}\right)}+\frac{[Mg^{2+}]}{[Mg^{2+}]_{ref}}e^{-\beta\left(E_{b_O}^{Mg^{2+}}-\mu_{Mg^{2+}_{ref}}\right)}\right)^4+e^{-\beta E_C}\left(1+\frac{[Ca^{2+}]}{[Ca^{2+}]_{ref}}e^{-\beta\left(E_{b_C}^{Ca^{2+}}-\mu_{Ca^{2+}_{ref}}\right)}+\frac{[Mg^{2+}]}{[Mg^{2+}]_{ref}}e^{-\beta\left(E_{b_C}^{Mg^{2+}}-\mu_{Mg^{2+}_{ref}}\right)}\right)^4}. \tag{S5}$$

Substituting variables in equation (S5):

$$K_{O0} = e^{-\beta(E_C - E_O)}\ , \quad \text{(S6)}$$

$$K_{ACa} = [Ca^{2+}]_{ref} e^{\beta\left(E_{b_C}^{Ca^{2+}} - \mu_{Ca^{2+}_{ref}}\right)}, \quad \text{(S7)}$$

$$K_{AMg} = [Mg^{2+}]_{ref} e^{\beta\left(E_{b_C}^{Mg^{2+}} - \mu_{Mg^{2+}_{ref}}\right)}, \quad \text{(S8)}$$

$$f_{Ca} = e^{-\beta\left(E_{b_C}^{Ca^{2+}} - E_{b_O}^{Ca^{2+}}\right)}, \quad \text{(S9)}$$

$$f_{Mg} = e^{-\beta\left(E_{b_C}^{Mg^{2+}} - E_{b_O}^{Mg^{2+}}\right)}\ . \quad \text{(S10)}$$

where $K_{Ax}$ stays for the respective equilibrium constants and $f_x$ for the respective allosteric factors of the ion $x$, we obtain $P_O$ in the absence of the RyR inhibition site:

$$P_O([Ca^{2+}],[Mg^{2+}]) = \frac{\left(1+\frac{[Ca^{2+}]}{f_{Ca}K_{ACa}}+\frac{[Mg^{2+}]}{f_{Mg}K_{AMg}}\right)^4}{\left(1+\frac{[Ca^{2+}]}{f_{Ca}K_{ACa}}+\frac{[Mg^{2+}]}{f_{Mg}K_{AMg}}\right)^4 + K_{O0}\left(1+\frac{[Ca^{2+}]}{K_{ACa}}+\frac{[Mg^{2+}]}{K_{AMg}}\right)^4}$$

$$= \frac{\left([Ca^{2+}]+f_{Ca}K_{ca}+\frac{f_{Ca}K_{ACa}}{f_{Mg}K_{AMg}}[Mg^{2+}]\right)^4}{\left([Ca^{2+}]+f_{Ca}K_{ACa}+\frac{f_{Ca}K_{ACa}}{f_{Mg}K_{AMg}}[Mg^{2+}]\right)^4 + K_{O0}f_{Ca}^4\left(K_{ACa}+Ca+\frac{K_{ACa}}{K_{AMg}}[Mg^{2+}]\right)^4}. \quad \text{(S11)}$$

Magnesium binding to the RyR inhibition site was treated as independent of the state of the RyR activation sites. This means that binding of $Mg^{2+}$ to the inhibition site renders the RyR channel non-conductive regardless of the state of the activation sites. Considering experimental data (Laver et al., 1997; Zahradnikova et al., 2003), the respective rate constant of $Mg^{2+}$ binding was set proportional to the square of magnesium concentration and the rate of $Mg^{2+}$ unbinding was set independent of magnesium concentration. This allows us to write an equation for $P_O$ of the full model in the form:

$$P_O([Ca^{2+}],[Mg^{2+}]) = \frac{{K_{IMg}}^2}{{K_{IMg}}^2+[Mg^{2+}]^2}\frac{\left([Ca^{2+}]+K_{ACa}f_{Ca}\left(1+\frac{[Mg^{2+}]}{K_{AMg}f_{Mg}}\right)\right)^4}{\left([Ca^{2+}]+K_{ACa}f_{Ca}\left(1+\frac{[Mg^{2+}]}{K_{AMg}f_{Mg}}\right)\right)^4 + K_{O0}f_{Ca}^4\left(Ca+K_{ACa}\left(1+\frac{[Mg^{2+}]}{K_{AMg}}\right)\right)^4}, \quad \text{(S12)}$$

where $K_{IMg}$ is the dissociation constant of $Mg^{2+}$ ions at the RyR inhibition site.

## 1.2 Construction of the Q-matrix of the RyR gating model

The presented model of RyR gating is a continuous-time Markov chain hence it is defined by a transition rate matrix $\boldsymbol{Q}$. This section describes the equations used for the construction of the Q-matrix (see Methods in the main text).

For the construction of the Q-matrix, the states $O_{ij0}$ are considered open states, and the non-conductive states $C_{ijk}$ and $O_{ij2}$ are considered closed states. The transition rates are written in Supplementary Table S1.

# 2 Supplementary Figures and Tables

## 2.1 Supplementary Tables

**Supplementary Table S1. Transition rates within the RyR gating model.**

| Transition | Rate |
|---|---|
| $Ca^{2+}$ binding to the activation site | |
| $C_{i,j,k} \rightarrow C_{i+1,j,k}, i + j < 4$ | $(4 - i - j) \cdot k_{AonCa} \cdot [Ca^{2+}]$ |
| $O_{i,j,k} \rightarrow O_{i+1,j,k}, i + j < 4$ | $(4 - i - j) \cdot k_{AonCa} \cdot \frac{[Ca^{2+}]}{f_{Ca}^{\lambda_{onCa}}}$ |
| $Ca^{2+}$ unbinding from the activation site | |
| $C_{i,j,k} \rightarrow C_{i-1,j,k}, i > 0$ | $i \cdot k_{AoffCa}$ |
| $O_{i,j,k} \rightarrow O_{i-1,j,k}, i > 0$ | $i \cdot k_{AoffCa} \cdot f_{Ca}^{1-\lambda_{onCa}}$ |
| $Mg^{2+}$ binding to the activation site | |
| $C_{i,j,k} \rightarrow C_{i,j+1,k}, i + j < 4$ | $(4 - i - j) \cdot k_{AonMg} \cdot [Mg^{2+}]$ |
| $O_{i,j,k} \rightarrow O_{i,j+1,k}, i + j < 4$ | $(4 - i - j) \cdot k_{AonMg} \cdot \frac{[Mg^{2+}]}{f_{Mg}^{\lambda_{onMg}}}$ |
| $Mg^{2+}$ unbinding from the activation site | |
| $C_{i,j,k} \rightarrow C_{i,j-1,k}, j > 0$ | $j \cdot k_{AoffMg}$ |
| $O_{i,j,k} \rightarrow O_{i,j-1,k}, j > 0$ | $j \cdot k_{AoffMg} \cdot f_{Mg}^{1-\lambda_{onMg}}$ |
| Transitions between O- and C-states | |
| $C_{i,j,k} \rightarrow O_{i,j,k}$ | $\frac{k_{CO}}{(f_{Ca}^{i} f_{Mg}^{j})^{\lambda_{CO}}}$ |
| $O_{i,j,k} \rightarrow C_{i,j,k}$ | $k_{OC}(f_{Ca}^{i} f_{Mg}^{j})^{1-\lambda_{CO}}$ |
| $Mg^{2+}$ binding/unbinding at the inhibition site | |
| $X_{i,j,0} \rightarrow X_{i,j,2}$ | $k_{IonMg}[Mg^{2+}]^2$ |
| $X_{i,j,2} \rightarrow X_{i,j,0}$ | $k_{IoffMg}$ |

**Supplementary Table S2. Mg-binding parameters used in this study.** Numbers in boldface represent the reference values estimated by fitting the model to data from the literature (see Methods).

| $k_{AonMg}$ ($\mu M^{-1}ms^{-1}$) | $k_{AoffMg}$ ($ms^{-1}$) | $f_{Mg}$ | | $k_{IonMg}$ ($10^{-7}\ \mu M^{-2}ms^{-1}$) | $k_{IoffMg}$ ($ms^{-1}$) |
|---|---|---|---|---|---|
| 0.000596 | 0.055 | 0.5 | 10 | 1.842 | 0.055 |
| 0.001107 | 0.102184 | 1 | 25 | 2.857 | 0.08529 |
| 0.002057 | 0.189846 | 1.56292535 | 40 | 3.871 | 0.11558 |
| 0.003822 | 0.352712 | 2.1258507 | 55 | 4.886 | 0.14587 |
| **0.0071** | **0.655298** | 2.68877605 | 70 | **5.9002** | **0.17616** |
| 0.009923 | 0.915828 | **3.2517014** | 85 | 8.6120 | 0.25712 |
| 0.013868 | 1.279939 | 4.93877605 | 100 | 11.323 | 0.33808 |
| 0.019381 | 1.788812 | 6.6258507 | 500 | 1.4035 | 0.41904 |
| 0.027087 | 2.5 | 8.31292535 | | 1.6746 | 0.5 |

## 3 References


Laver, D.R., Baynes, T.M., and Dulhunty, A.F. (1997). Magnesium inhibition of ryanodine-receptor calcium channels: Evidence for two independent mechanisms. *J Membr Biol* 156**,** 213-229.

Marzen, S., Garcia, H.G., and Phillips, R. (2013). Statistical mechanics of Monod-Wyman-Changeux (MWC) models. *J Mol Biol* 425(9)**,** 1433-1460. doi: 10.1016/j.jmb.2013.03.013.

Zahradnikova, A., Dura, M., Gyorke, I., Escobar, A.L., Zahradnik, I., and Gyorke, S. (2003). Regulation of dynamic behavior of cardiac ryanodine receptor by Mg2+ under simulated physiological conditions. *Am J Physiol* 285(5)**,** C1059-1070.
